# Exploring the therapeutic potential of Sirt6-enriched adipose stem cell-derived exosomes in myocardial ischemia–reperfusion injury: unfolding new epigenetic frontiers

**DOI:** 10.1186/s13148-023-01618-2

**Published:** 2024-01-03

**Authors:** Kun Liu, Hecheng Wang, Yiou Wang, Xiaoxu Zhang, Ruihu Wang, Zhaoxuan Zhang, Jian Wang, Xinran Lu, Xiaoyu Wu, Yanshuo Han

**Affiliations:** 1 Department of Cardiac Surgery, Affiliated Hospital, Guizhou Medical University, Guiyang, China; 2 School of Life and Pharmaceutical Sciences, Dalian University of Technology, Panjin, China; 3 Department of Anesthesiology, General Hospital of Northern Theater Command, Shenyang, China; 4 Department of Vascular Surgery, Shanghai Ninth People’s Hospital, Shanghai Jiao Tong University School of Medicine, Shanghai, China

**Keywords:** Sirtuin 6, Adipose stem cell-derived exosomes, Myocardial ischemia–reperfusion injury, Mitophagy, Pyroptosis, Anoxia-reoxygenation, AIM2 inflammasome

## Abstract

**Background:**

The management of myocardial ischemia–reperfusion injury (MIRI) presents continuous therapeutic challenges. NAD-dependent deacetylase Sirtuin 6 (Sirt6) plays distinct roles in various disease contexts and is hence investigated for potential therapeutic applications for MIRI. This study aimed to examine the impact of Sirt6-overexpressing exosomes derived from adipose stem cells (S-ASC-Exo) on MIRI, focusing on their influence on AIM2-pyroptosis and mitophagy processes. The sirtuin family of proteins, particularly Sirtuin 6 (Sirt6), play a pivotal role in these processes. This study aimed to explore the potential therapeutic effects of Sirt6-enriched exosomes derived from adipose stem cells (S-ASC-Exo) on regulating MIRI.

**Results:**

Bioinformatic analysis revealed a significant downregulation of Sirt6 in MIRI subjected to control group, causing a consequential increase in mitophagy and pyroptosis regulator expressions. Therefore, our study revealed that Sirt6-enriched exosomes influenced the progression of MIRI through the regulation of target proteins AIM2 and GSDMD, associated with pyroptosis, and p62 and Beclin-1, related to mitophagy. The introduction of S-ASC-Exo inhibited AIM2-pyroptosis while enhancing mitophagy. Consequently, this led to a significant reduction of GSDMD cleavage and pyroptosis in endothelial cells, catalyzing a deceleration in the progression of atherosclerosis. Extensive in vivo and in vitro assays were performed to validate the expressions of these specific genes and proteins, which affirmed the dynamic modulation by Sirt6-enriched exosomes. Furthermore, treatment with S-ASC-Exo drastically ameliorated cardiac functions and limited infarct size, underlining their cardioprotective attributes.

**Conclusions:**

Our study underscores the potential therapeutic role of Sirt6-enriched exosomes in managing MIRI. We demonstrated their profound cardioprotective effect, evident in the enhanced cardiac function and attenuated tissue damage, through the strategic modulation of AIM2-pyroptosis and mitophagy. Given the intricate interplay between Sirt6 and the aforementioned processes, a comprehensive understanding of these pathways is essential to fully exploit the therapeutic potential of Sirt6. Altogether, our findings indicate the promise of Sirt6-enriched exosomes as a novel therapeutic strategy in treating ischemia–reperfusion injuries and cardiovascular diseases at large. Future research needs to underscore optimizing the balance of mitophagy during myocardial ischemia to avoid potential loss of normal myocytes.

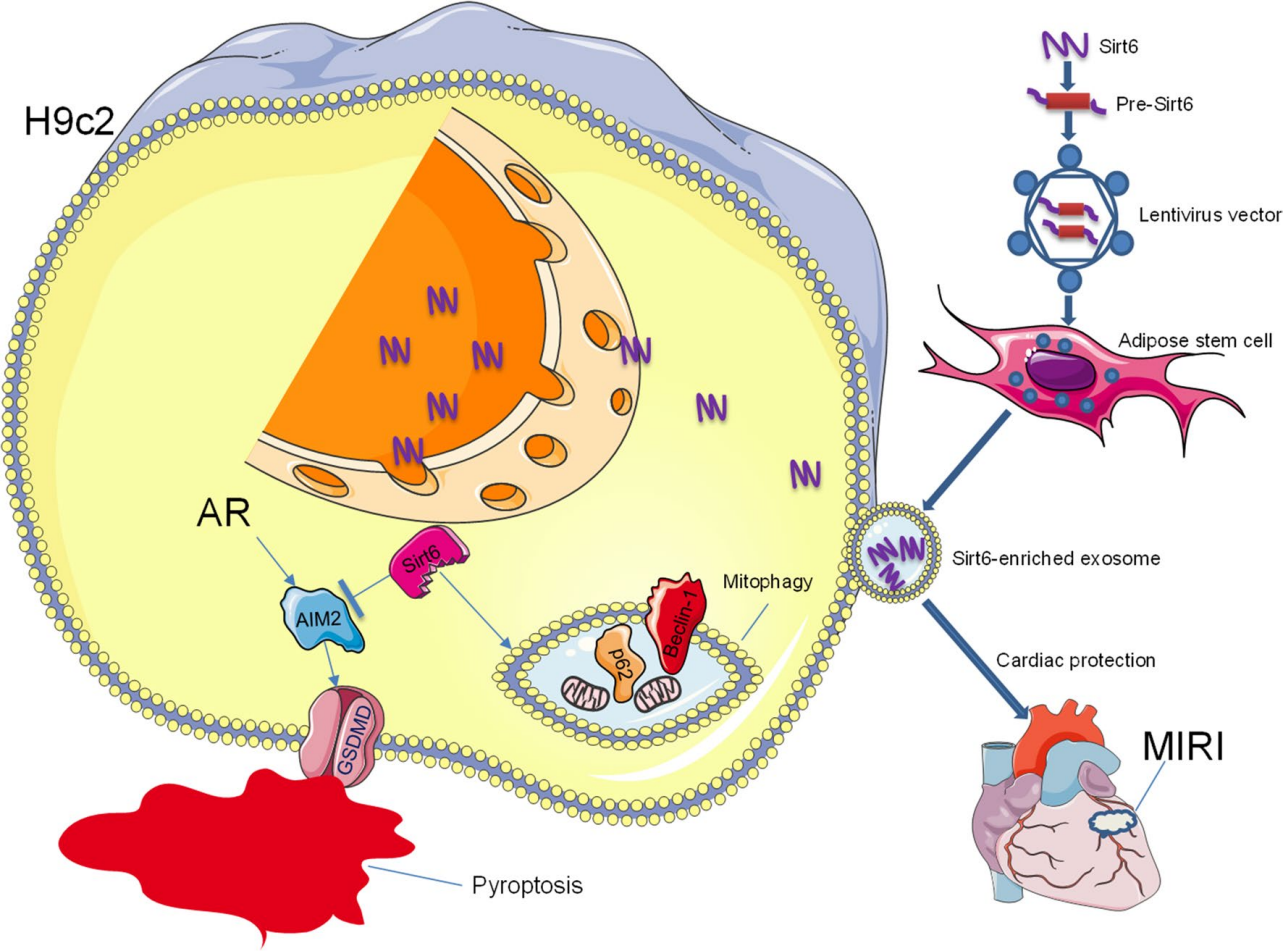

**Supplementary Information:**

The online version contains supplementary material available at 10.1186/s13148-023-01618-2.

## Introduction

Despite the great progress of endovascular technique, coronary artery bypass grafting (CABG), especially off-pump CABG (OPCAB), is still the best choice for coronary heart disease (CHD), due to its encouraging long-term patency rate [1]. Maintaining no-blood surgical field is necessary for fine anastomosis of vessels, so coronary arteries need to be occluded temporarily. A series of post-OPCAB complications induced by MIRI, such as low output syndrome, myocardial infarction, and heart failure, remain understood incompletely and not entirely preventable [2]. The principal molecular mechanism triggered by MIRI involves oxidative stress and subsequent excessive production of reactive oxygen species (ROS), which disrupts the balance of repair and clearance of large protein aggregates or damaged organelles, resulting in activation of cell death pathways [3].

Mitophagy, specialized clearance of impaired mitochondria, plays a crucial role in alleviating cell injury before dysfunctional mitochondria cause activation of cell death [4]. In a mouse model of diabetes, activation of GLP-1R has been shown to alleviate depressed behaviors by decreasing the level of ROS to improve mitochondrial functions and promote mitophagy [5]. In retinal pigment epithelium exposed to hydrogen peroxide, DNA damage and apoptosis are associated with inhibited mitophagy, which could be reversed by downregulating the level of ROS [6]. Collectively, available findings suggest that enhancing mitophagy exhibits beneficial outcomes via blocking of ROS production and accumulation. Pyroptosis, an inflammasome-induced programmed cell death [7], is considered to influence various pathophysiological responses in multifaceted ways, including oxidative stress [3, 7]. In recent decades, the oxidative stress-induced pyroptosis has been widely studied in various diseases, especially in cerebral ischemia [8], atherosclerosis [9], and cancer treatment [10]. GSDMD is a pore-forming protein, activated by inflammasome complexes, located on the cell membrane, and finally mediates the release of inflammatory cytokines and pyroptotic death [11]. AIM2 was indicated to be notably associated with pyroptosis, typically by activating caspase-1/GSDMD signaling [12]. In our previous study, convincing evidence indicates that inhibition of NLRP3-inflammasome-pyroptosis conducts reendothelialization effect against mechanical injury to endarterium, and the most pivotal modulatory node is ROS [13]. These fundamental researches render mitophagy and pyroptosis regulatory targets for the prevention and treatment of MIRI.

Sirt6 belongs to NAD-dependent deacetylase family and participates in the regulations of important cellular processes, including cellular proliferation, aging, DNA damage repair, mitochondrial function, and inflammation [14]. Sirt6 has been found to directly act on mitochondria, inhibit ROS accumulation, and then suppress cellular senescence [15]. Nevertheless, the regulatory mechanism of Sirt6/mitophagy/AIM2-pyroptosis in MIRI is yet to determined. Exosomes are extracellular vesicles that are released by almost all types of cells, deliver various bioactive molecules, and mediate intracellular communications [16]. They carry a variety of biomolecules, including proteins, lipids, cytokines, and nucleic acids such as mRNA, microRNA, and other non-coding RNAs [17]. These biomolecules reflect the functional and biochemical characteristics of their originating cells. Sirt6 is a NAD + -dependent deacetylase with well-established roles in cellular processes, such as metabolism, DNA repair, and inflammation. Recent studies have highlighted the significance of Sirt6 in cardiovascular diseases and MIRI due to its antioxidative, anti-inflammatory, and anti-apoptotic properties. Mesenchymal stem cells (MSC) are confirmed to have multipotential plasticity and more likely to secrete large amounts of exosomes [18]. With the continuous study of exosome, the function of exosomes could be enhanced by genetic modification of MSCs to transport particular proteins [18].

In this study, we delivered Sirt6 to cardiomyocytes via exosomes derived from ASCs that were genetically modified to overexpress Sirt6 and investigated the regulatory mechanisms of Sirt6/mitophagy/AIM2-pyroptosis in cardiomyocytes subjected to anoxia/reoxygenation (AR). By transfecting adipose stem cells with Sirt6, we aimed to increase the therapeutic potential of secreted exosomes to confer cardioprotection during MIRI. To evaluate the role of mitophagy in MIRI, p62 and Beclin-1 as essential proteins of conjugation system were measured. We highlight the cardioprotective effect of Sirt6-modified exosomes on ischemic myocardia, which may provide a novel prospect for the prevention and treatment of MIRI.

## Methods

### Bioinformatics analysis

To investigate gene expression changes in young and aged wild-type (WT) mice undergoing myocardial ischemia–reperfusion injury, we performed expression profiling using high-throughput sequencing (GSE130217). The experimental design involved two groups: Young (3–4 months) C57BL/6 J mice (young-WT) and aged (24–26 months) C57BL/6 J mice (aged-WT), with six mice in each group. The surgical procedure commenced with a left lateral thoracotomy to expose the heart. Subsequently, the left anterior descending coronary artery (LAD) was occluded for 45 min using an 8–0 nylon suture, and polyethylene tubing was utilized to prevent arterial injury. Reperfusion was initiated and continued for 24 h. After the reperfusion period, myocardial tissue samples were collected from both young-WT plus aged-WT mice and MIRI young-WT plus aged-WT (*n* = 6 per group). High-throughput sequencing was conducted using a next-generation sequencing platform, such as Illumina HiSeq, to generate sequence reads. Differential expression analysis was conducted using statistical methods, such as the R software package "limma," to identify genes with significantly altered expression between young-WT and aged-WT mice. Genes with an adjusted P value < 0.05 and an absolute log fold change (|logFC|) greater than or equal to 0.5 were considered differentially expressed genes (DEGs). Common Sirt family regulators include seven regulators (Sirt1, Sirt2, Sirt3, Sirt4, Sirt5, Sirt6, and Sirt7, Additional file 1). Thereafter, the Sirt family regulators with P value < 0.05 and |logFC|≥ 1.0 were considered as differentially expressed Sirt family genes (DeSFGs).

Thirty mitophagy-related genes were downloaded from the Pathway Unification database (https://pathcards.genecards.org/), listed in Additional file 2. For the selection of pyroptosis-related genes (PRGs), we employed a multi-step approach. Firstly, we extracted PRGs from the GeneCards database (https://www.genecards.org/). Specifically, we retrieved genes with relevance scores greater than 7, resulting in a selection of six genes. To expand our list of PRGs, we obtained a set of 27 pyroptosis-related genes (Reactome pyroptosis) from the Molecular Signature Database v7.4 (MSigDB). Additionally, we identified 33 pyroptosis genes from relevant literature sources [19, 20]. To ensure non-redundancy in our gene set, we removed any overlapping genes among the three sources mentioned above. This process yielded a final set of 51 PRGs, which were considered for further investigation in our study. By employing this comprehensive approach, we aimed to capture a diverse range of PRGs from multiple reliable sources, enhancing the robustness and coverage of our analysis (Additional file 3). These PRGs will serve as valuable candidates for exploring the mechanisms and implications of pyroptosis in our research.

### The network construction of Sirts between mitophagy-related genes and pyroptosis-related genes

The above Sirt family genes were crossed with the differential mitophagy-related genes and pyroptosis-related genes to obtain the overlapped genes. The network of association between DeSFGs and PRGs and MRGs was constructed. The PPI network was analyzed for the overlapped genes employing the Search Tool for the Retrieval of Interacting Genes (STRING) database (https://www.string-db.org) with a combined score > 0.4. Subsequently, two networks were visualized by the Cytoscape software.

### Immune infiltration analysis using CIBERSORTx

To conduct immune infiltration analysis of GSE130217, we utilized CIBERSORTx (https://cibersortx.stanford.edu). The leukocyte signature matrix was employed as the signature gene file, enabling the identification of 13 distinct human hematopoietic cell phenotypes. By applying this approach, we obtained the proportions of immune cells in each sample. Subsequently, we analyzed the relationship between immune cells and DeSFGs.

### Animals and acquisition and preparation of rat adipose stem cells

Adipose-derived Stem Cells (ASCs) were procured from male Sprague Dawley (SD) rats weighing approximately 250 g. In this study, we used inbred Sprague Dawley rats to ensure genetic consistency among the experimental subjects. This well-established model is broadly recognized for its predictable and uniform responses to ischemia, comprehensively minimizing the potential variability that could arise from genetic differences. Moreover, Sprague Dawley rats are known for their large size and, hence an increased tolerance to repeated blood samples and test substance administrations, making them an ideal model for myocardial ischemic-reperfusion studies of this nature. However, it is also essential to note that we saw uniform responses to ischemia in all rats. Repeated measures were taken throughout the study to ensure comparable ischemic conditions across the subjects. These rats were chosen specifically for this study and their use was permitted by the Ethics Review Committee of Animal Experimentation of Guizhou Medical University, under approval number AEEIF2200612. The process of ASC acquisition and preparation began with the isolation of rat adipose tissues [21, 22]. Following rat euthanasia under ethically approved protocols, adipose tissues were collected, processed, and subjected to a series of enzymatic digestion and centrifugation steps to separate the stromal vascular fraction, which contains the ASCs. The isolated ASCs were then cultured in an appropriate growth medium, the composition of which was optimized to encourage the proliferation and stem cell characteristics of the ASCs. The cells were maintained under controlled conditions in a standard cell culture incubator set at 37 °C with 5% CO_2_. Subsequently, a transfection procedure was performed. The ASCs were transfected with specific genes using recombinant plasmids obtained from Genechem Biosciences Inc (Shanghai, China). This procedure was optimally designed to enable the genes to be successfully incorporated and expressed in the rat ASCs.

### Culture and transfection of ASCs

Rat stem cells were obtained from Genechem Biosciences Inc (Shanghai, China). The cells were cultured in rat adipose stem cell (ASC) basal medium supplemented with 10% fetal bovine serum and 1% penicillin–streptomycin. Then, they were transfected with rat Sirt6 lentiviral gene transfer vectors, also constructed by Genechem Biosciences Inc. The Sirt6 lentiviral vectors used in this study were designed specifically for overexpression of the Sirt6 gene. This vector system not only allows for efficient delivery and stable integration of the Sirt6 gene sequence into the host cell genome, but also provides robust and long-term expression of Sirt6. As part of this system, the Sirt6 gene is under the control of a cytomegalovirus (CMV) promoter, which ensures high-level and persistent expression in the host cells. In addition to the Sirt6 gene, these lentiviral vectors also carry a green fluorescent protein (GFP) sequence. GFP is expressed as a fusion protein with Sirt6, allowing for easy visualization of Sirt6 expression in transduced cells. The GFP tag does not interfere with the Sirt6 functionality because it is coupled via a flexible linker sequence that allows both proteins to fold and function independently. The vector also contains a neomycin resistance gene to enable the selection of stably transfected cells using the antibiotic G418. During the transfection process, the cells were exposed to the lentiviral particles at a multiplicity of infection of 10. Following 24 h of transduction, the virus-containing medium was replaced with the regular culture medium. The cells were then incubated for an additional 48 h to allow for the integration and expression of the Sirt6 gene. The successful transduction was confirmed by monitoring GFP expression using fluorescence microscopy. Post the 48-h incubation period, these cells were ready for subsequent experimental applications, such as protein-specific assays or studies addressing the effects of Sirt6 overexpression.

### Exosome isolation and characterization

After 48 h culture of Sirt6-ACSs (S-ASC), the exosomes were isolated according to the kit instructions. Exosomes were isolated from the conditioned medium of adipose stem cells (ASCs) using the ExoQuick-TC™ Exosome Precipitation Solution (System Biosciences, Palo Alto, CA, USA) according to the manufacturer's instructions. Briefly, the conditioned medium was centrifuged at 3000 g for 15 min to remove cells and debris. The supernatant was then transferred to a new tube and mixed with the ExoQuick-TC™ solution. The mixture was incubated at 4 °C overnight to allow for exosome precipitation. The following day, the mixture was centrifuged at 1500 g for 30 min to pellet the exosomes. The supernatant was discarded, and the exosome pellet was resuspended in 1 × phosphate-buffered saline (PBS) for further analysis. Ultracentrifugation method was used for exosome isolation. In these cases, the conditioned medium was first centrifuged at 300 g for 10 min to remove cells, followed by centrifugation at 2000 g for 20 min to remove debris. The supernatant was then filtered through a 0.22 µm filter to remove larger vesicles. The filtered supernatant was ultracentrifuged at 100,000 g for 70 min to pellet the exosomes. The exosome pellet was washed with PBS and ultracentrifuged again at 100,000 g for 70 min. The final exosome pellet was resuspended in PBS for further analysis. The morphologic characteristics of exosome were observed using transmission electron microscopy. Expressions of CD9 (20,597–1-AP, 1:3000 incubated, Proteintech Group, Inc. Rosemont, IL, USA), CD63 (67605-1-Ig, 1:10,000 incubated, Proteintech Group, Inc. Rosemont, IL, USA), CD81 (abs113678, 1:5000 incubated, Absin Bioscience Inc. Shanghai, China), and Calnexin (abs145735, 1:4000 incubated, Absin Bioscience Inc. Shanghai, China) were detected by western blot.

### Tracking of exosomes engulfed by cardiomyocytes

Rat cardiomyocytes (H9c2) were purchased from FudanCell (FH1004, FuHeng BioLogy, Shanghai, China) and the culture medium was then replaced with 1 mL of fresh Dulbecco's Modified Eagle Medium (DMEM) containing 10% Fetal Bovine Serum (FBS) and 1% penicillin–streptomycin. Before the incubation, the exosomes were labeled with PHK67 membrane dye (Sigma-Aldrich, St. Louis, MO, USA) according to the manufacturer's instructions. The labeled exosomes were then resuspended in 1 × phosphate-buffered saline (PBS). For the incubation, H9c2 cells were seeded in a 24-well plate and allowed to adhere overnight. Subsequently, 200 µL of the PHK67-labeled exosome suspension was added to each well. The cells were incubated with the labeled exosomes for 24 h at 37 °C in a humidified incubator with 5% CO_2_ for tracking purposes. We established the anoxia/reoxygenation (AR) conditions following a robust methodology. The incubation of H9c2 cells in an oxygen-free and low-glucose culture medium occurred inside an anaerobic workstation (Coy anaerobic chamber), which ensured the complete absence of ambient oxygen. This specialized equipment creates a controlled environment with low oxygen levels, allowing for precise manipulation of oxygen concentrations during the experiment. Post-incubation, cells were immediately transferred to a normoxic and high-glucose environment. This rapid transition simulated in vivo conditions of reperfusion after ischemic injury. After incubation with PHK67-labeled exosomes, the H9c2 cells were washed three times with phosphate-buffered saline (PBS) to remove any unbound exosomes. The cells were then fixed with 4% paraformaldehyde for 15 min at room temperature. After fixation, the cells were washed again with PBS and then mounted on glass slides using a mounting medium containing DAPI (4',6-diamidino-2-phenylindole) to stain the nuclei. The engulfment of exosomes by H9c2 cells was visualized using a confocal laser scanning microscope (Leica TCS SP8, Leica Microsystems, Wetzlar, Germany). The PHK67-labeled exosomes were excited at 490 nm and emission was collected at 502 nm. The DAPI-stained nuclei were excited at 358 nm and emission was collected at 461 nm. The microscope settings were kept consistent across all samples to allow for comparison. The images were processed and analyzed using the Leica Application Suite X (LAS X) software.

## Experimental protocol

**Part I.** Evaluation of the cardioprotective effect of S-ASC-Exo.

**In vitro**. MTT assay and LDH release: (1) Sham: PBS (2 µL) without AR, (2) Control: PBS (2 µL), (3) ASC-Exo: ASC-Exo (2 µL, 2 µg µL^−1^), and (4) S-ASC-Exo: S-ASC-Exo (2 µL, 2 µg µL^−1^) was respectively added to the 6-well plate cultured with H9c2s (1 × 10^5^/well) subjected to AR. In a parallel series of experiments, H9c2s were collected from the 4 groups to determine the expressions of AIM2-pyroptosis and mitophagy.

**In vivo**. (1) Sham (*n* = 6): PBS (200 µL) without MIRI, (2) Control (*n* = 6): PBS (200 µL), (3) S-ASC-Exo (*n* = 6): S-ASC-Exo (200 µL, 2 µg µL^−1^) was respectively injected 1 h before MIRI. All rats received echocardiography, and myocardial tissues were harvested for HE staining, Masson trichrome staining, and Evans blue staining. In a parallel series of experiments, myocardial tissues were harvested from the 3 groups (*n* = 6) to determine the expressions of AIM2-pyroptosis and mitophagy.

**Part II.** Reversion of the cardioprotective effect S-ASC-Exo using siSirt6.

**In vitro**. MTT assay and LDH release: (1) Control: PBS (2 µL), (2) S-ASC-Exo: S-ASC-Exo (2 µL, 2 µg µL^−1^), (3) siSirt6: siSirt6 (2 µL), and (4) S-Exo + siSirt6: S-ASC-Exo (2 µL, 2 µg µL^−1^) + siSirt6 (2 µL) was respectively added to the 6-well plate cultured with H9c2s (1 × 10^5^/well) subjected to AR. In a parallel series of experiments, H9c2s were collected from the 4 groups to determine the expressions of AIM2-pyroptosis and mitophagy.

**Part III.** Further verify the pathways of pyroptosis and mitophagy.

**In vitro**. MTT assay and LDH release: (1) Control: PBS (2 µL), (2) siSirt6: siSirt6 (2 µL), (3) VX-765 (inhibitor of Caspase-1): VX-765 (2 µL), and (4) siSirt6 + VX-765: siSirt6 (2 µL) + VX-765 (2 µL) was, respectively, added to the 6-well plate cultured with H9c2s (1 × 10^5^/well) subjected to AR. In a parallel series of experiments, H9c2s were collected from the 4 groups to determine the expressions of Caspase-1 and GSDMD.

**In vitro**. The MTT assay and LDH release: (1) Control: PBS (2 µL), (2) S-ASC-Exo: S-ASC-Exo (2 µL, 2 µg µL^−1^), (3) 3-MA (3-methyladenine, inhibitor of mitophagy): 3-MA (2 µL), and (4) S-ASC-Exo + 3-MA: S-ASC-Exo (2 µL, 2 µg µL^−1^) + 3-MA (2 µL) was respectively added to the 6-well plate cultured with H9c2s (1 × 10^5^/well) subjected to AR. In a parallel series of experiments, H9c2s were collected from the 4 groups to determine the expressions of mitophagy.

### Sirt6 silencing

Small interfering RNA (siRNA) targeting Sirt6 was purchased from Lipofectamine 3000 (Invitrogen, Carlsbad, CA, USA). The H9c2s (1 × 10^5^/well) were washed with PBS and transfected with siSirt6, or non-targeting siRNA using lipofectamine 3000 (Invitrogen, Carlsbad, CA, USA) according to the manufacturer’s protocol. After 6 h incubation, the medium was replaced with rat MSC basal media for 48 h incubation. As a control for the Sirt6 silencing experiments, a non-targeting siRNA (also known as a scramble siRNA) was used. This scramble siRNA was purchased from Dharmacon (Lafayette, CO, USA) and is designed to not target any known mammalian genes, thereby serving as a negative control to account for any non-specific effects of the transfection process. The transfection with the scramble siRNA was performed in parallel with the Sirt6 siRNA transfection. Briefly, H9c2 cells were seeded in a 24-well plate and allowed to reach 70–80% confluence. The cells were then transfected with the scramble siRNA using Lipofectamine 3000 (Invitrogen, Carlsbad, CA, USA) according to the manufacturer's instructions. The final concentration of the scramble siRNA used was 50 nM, equivalent to the concentration of the Sirt6 siRNA. Post-transfection, the cells were further incubated for 48 h before being harvested for downstream analysis. The efficiency of the transfection and the absence of off-target effects were assessed by comparing the results from the scramble siRNA-transfected cells with those from the Sirt6 siRNA-transfected cells and the non-transfected cells. qRT-PCR and western blot were performed to evaluate the knockdown efficiency.

### In vitro cardiomyocyte anoxia/reoxygenation model

In vitro rat cardiomyocyte anoxia/reoxygenation (AR) was established by incubating H9c2s in oxygen-free (95% N_2_ and 5% CO_2_), low-glucose culture medium for 2 h and transferred to normal oxygen level and high-glucose culture medium for 1 h.

### MTT assay

The viability of H9c2s was assessed via MTT assay. H9c2s (1 × 10^3^/well) were plated into 96-well plates and treated with PBS, AR, ASC-Exo, S-ASC-Exo, siSirt6, 3-MA, and VX-765 as indicated in each experiment. At the corresponding time, 20 mL of MTT (5 mg/mL) (Roche, Basel, Switzerland) was added to each well. The culture medium was discarded after 4 h reaction, and 150 mL of DMSO was added to each well and incubated for 15 min. The absorbance value at a wavelength of 490 nm was recorded.

### Enzyme-linked immunosorbent assay for LDH

The lactate dehydrogenase (LDH) levels in the cell culture supernatants were determined using the LDH Cytotoxicity Assay Kit (Beyotime Institute of Biotechnology, Shanghai, China) according to the manufacturer's instructions. Briefly, 120 µL of cell culture supernatant was transferred to a 96-well plate, followed by the addition of 60 µL of LDH reaction mixture. The plate was then incubated at room temperature for 30 min in the dark. The reaction was stopped by adding 60 µL of stop solution. The absorbance was measured at 490 nm using a microplate reader. The LDH activity in the supernatant, which is directly proportional to the absorbance, was then calculated. This activity serves as an indicator of cell membrane integrity and is used to assess the level of cell damage or cytotoxicity. Lactate dehydrogenase (LDH) release was used as an additional marker of cell viability in our study. LDH is a cytoplasmic enzyme that is rapidly released from cells upon plasma membrane damage or rupture, making it a well-established marker for assessing cell membrane integrity and cytotoxicity. However, it is worth noting that while LDH release is a reliable marker, it mainly indicates cell damage or necrosis, rather than distinguishing between viable and non-viable cells. Therefore, in our study, we combined both LDH and MTT assays to obtain a comprehensive assessment of cell viability and damage.

### Myocardial ischemia/reperfusion model and tail vein injection

Prior to the surgical procedure, the rats were anesthetized using a combination of ketamine (80 mg/kg) and xylazine (10 mg/kg), administered intraperitoneally. The depth of anesthesia was monitored by assessing the pedal withdrawal reflex and adjusting the dose of anesthetic as necessary. Post-surgical analgesia was provided to minimize discomfort and pain. Buprenorphine (0.05 mg/kg) was administered subcutaneously every 12 h for the first 48 h following the surgery. All procedures were performed in accordance with the guidelines for the care and use of laboratory animals and were approved by the Institutional Animal Care and Use Committee. Every effort was made to minimize the number of animals used and their suffering. After satisfactory anesthesia, the limbs of rat were connected to electrocardiogram. The chest wall was opened to expose heart, and the left anterior descending coronary artery (LAD) was ligated with 6–0 Prolene suture. The use of the rat model and the 30-min ligation is a widely accepted and reliable method for inducing MIRI, as demonstrated in previous study [23]. To confirm effective ischemia and reperfusion, we monitored alterations in the EKG, an established marker of reperfusion. The successful occlusion was indicated by a typical ST segment elevation and then the successful restoration marked by a recovered ST segment. The occlusion was sustained for 30 min, and then the blood supplement was restored. After the ligation of the left anterior descending (LAD) coronary artery, some blood loss occurred. To compensate for this loss and maintain normal circulatory function, an isotonic saline solution was administered to the rats. Specifically, a volume of isotonic saline approximately equal to the estimated volume of blood loss was slowly injected into the rat's tail vein immediately after the surgical procedure. This helped to restore the blood volume, maintain the blood pressure, and ensure the proper perfusion of organs and tissues. Rats in the Sham group received thoracotomy without LAD occlusion. The administration of exosomes was performed through tail vein injection.

### Echocardiographic measurement

Echocardiography was conducted 3 days after surgery. The ligation of the left anterior descending (LAD) coronary artery was performed to induce myocardial infarction. The chest was reopened, and the suture was tightened to reocclude the LAD. Evans blue dye was then injected into the left ventricular cavity to delineate the AAR. The heart was then excised and sliced into 2-mm-thick sections. The slices were incubated in 1% triphenyl tetrazolium chloride (TTC) to stain viable myocardium. The AAR was identified as the area not stained by Evans blue, and the infarct size was identified as the area not stained by TTC within the AAR. The ultrasound Vevo 770 (Toronto, Ontario, Canada) was operated to record ejection fraction (EF%) and fractional shortening (FS%).

### Evaluation of area at risk and infract size

On the 3rd day after surgery, the LADs in all groups were ligated. 1% Evans Blue was infused into the ascending aorta to delineate the ischemic (area at risk, AAR) from the non-ischemic areas. The heart was cut into three transverse slices from heart apex to the ligation site. All the slices were incubated in 1% triphenyl tetrazolium chloride (TTC) to make differentiation between the necrotic (pale) and viable (brick red) areas. ImageJ software was used to calculate AAR/ left ventricle × 100% and infarct size (IS) /AAR × 100%.

### Histological study

On the 3th day after surgery, the hearts were harvested for histological study. Myocardial tissues were routinely dehydrated and embedded, and cut into slices with a thickness of 5 μm. The slices were dewaxed with xylene, dehydrated with gradient ethanol solution, then stained with hematoxylin for 8 min followed by the differentiation with hydrochloric acid ethanol solution, stained with eosin for 12 min, and dehydrated with gradient ethanol solution. Finally, the slices were sealed using neutral gum. Hematoxylin–eosin (HE) staining was performed to observe the morphologic changes of cardiomyocytes.

The slices were stained with celestine blue for 3 min, with Mayer's hematoxylin for 3 min, and with acid ethanol differentiation solution for 3 min. After that, the slices were washed with water for 30 min, stained with fuchsin for 10 min, with molybdophosphoric acid for 10 min, and with aniline blue for 5 min. The aniline blue was rinsed off with weak acid solution, weak acid solution was added again to cover the slices for 3 min. Finally, the slices were sealed using neutral gum.

Masson staining: Myocardial tissue sections were deparaffinized and rehydrated, the sections were stained with Masson's trichrome solution, comprising hematoxylin, Biebrich scarlet-acid fuchsin, and aniline blue. After staining, the sections underwent differentiation and dehydration steps. Finally, the stained sections were mounted for microscopic examination. This staining method allows for the differentiation of collagen fibers (blue) from other tissue components, providing a qualitative and quantitative assessment of fibrosis. All histological assessments were conducted by an experienced pathologist.

### Transmission electron microscopy

The specimens were mixed with 4% paraformaldehyde and then moved onto a carbon-coated copper grid. The membranes were absorbed for 30 min. Subsequently, the specimens were washed and fixed using 100 mL 1% glutaraldehyde, and then were contrasted with 4% uranyl acetate (UA) for 5 min. Finally, the specimens were embedded in a mixture of 4% UA and 2% methylcellulose solution. The transmission electron microscopy (JEM-2100 JEOL, Tokyo, Japan) was used.

### Quantitative real time polymerase chain reaction

Total RNAs of cells and exosomes were isolated with Trizol reagent (Invitrogen, Carlsbad, USA). RNAs were reverse transcribed to cDNAs using PrimeScript RT reagent Kit (Takara, Osaka, Japan). qRT-PCR was conducted using SYBR Green Supermix Kit (BD, USA). GAPDH was used as internal control for normalization of mRNAs. Gene expression levels were quantified by 2^*−ΔΔCT*^ method, the corresponding primer sequences in (Table 1).

**Table 1:** Primer sequences

Rat	Forward primer	Reverse primer
Sirt6	GGCAGTCATTGTCTCCACCA	TCTCGAAGGTGGTGTCAAAC
GAPDH	GCCACATCGCTCAGACACC	AATCCGTTGACTCCGACCTTC
AIM2	GTCCTCAAGCTAAGCCTCAGA	CACCGTGACAACAAGTGGAT
GSDMD	ATGCCATCGGCCTTTGAGAAA	AGGCTGTCCACCGGAATGA
p62	ACAGCCCAAACGTGCAGTAA	CTGATGCGGAACTACATCTGAAT
Beclin-1	TGGGGAGGTTAGGATTTGGGA	GAGCCGTAGGGTGGAAAGC

### Western blot

The cells, exosomes, and myocardial tissues were digested with RIPA. RIPA (radioimmunoprecipitation assay) lysis buffer used for protein extraction is: 50 mM Tris–HCl (pH 7.4), 150 mM NaCl, 1% Triton X-100, 0.5% sodium deoxycholate, 0.1% SDS (Sodium dodecyl sulfate), and 1 mM EDTA. Just before use, protease and phosphatase inhibitors are added, including 1 mM PMSF (phenylmethylsulfonyl fluoride), 1 µg/mL each of Aprotinin, Leupeptin, and Pepstatin, and 1 mM each of Na3VO4 (sodium orthovanadate) and NaF (sodium fluoride). All reagents are from Sigma-Aldrich (St. Louis, MO, USA). Protein concentrations were determined using spectrophotometer (Beckman, CA, USA) and adjusted by adding RIPA buffer. Equivalent amounts of protein were separated by polyacrylamide gel electrophoresis and transferred to PVDF membranes by electroblotting. Then the membranes were blocked in 5% BSA blocking buffer for 1 h. After blocking, the membranes were incubated with proportionally diluted primary anti-Sirt6, anti-CD9, anti-CD63, anti-CD81, anti-Calnexin, anti-AIM2, anti-GSDMD, anti-Caspase-1, anti-p62, and anti-Beclin-1 antibodies (Abcam, MA, USA) at 4℃ overnight. The membranes were washed with TBST for three times and incubated with diluted secondary antibodies for 1 h. The membranes were washed with TBST for another three times. Lastly, the membranes were incubated with ECL working solution and soon detected using a chemiluminescence system. The protein expressions were analyzed with ImageJ software and quantified as a relative fold to the sham group or the control group after normalization with GAPDH.

### Statistical analysis

Each experiment was repeated three times at least. Statistical analysis was performed using SPSS 25.0. The Shapiro–Wilk test was performed to confirm whether data belonged to normal distribution. Parametric values were analyzed by one-way ANOVA followed by Bonferroni correction for post hoc testing. Nonparametric values were analyzed by Kruskal–Wallis test with Dunn-Bonferroni correction for post hoc testing correction. The relationships between immune cells were determined using Pearson correlation analysis. *P* < 0.05 was considered statistically significant.

## Results

### Bioinformatics analysis

**Fig. 1:**
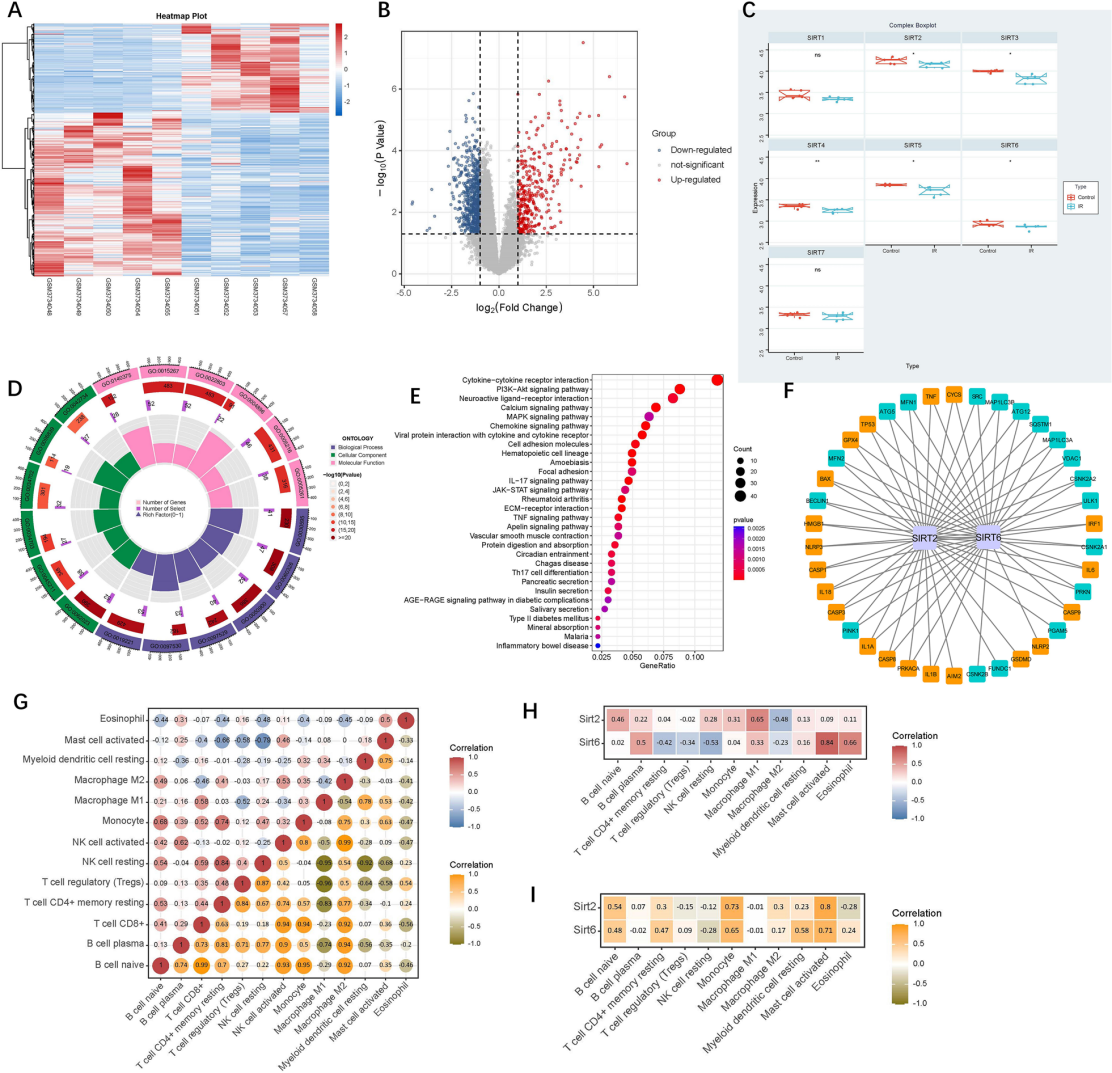
Differential gene expression analysis heat map (**A**), volcano plot (**B**), regulating expression level of Sirt family regulators (**C**) from GSE130217 database; The GO enrichment analysis (**D**) and KEGG pathway enrichment analysis (**E**) for differential expression genes (DEGs); Building the network between Sirt2 and Sirt6 and mitophagy and pyroptosis-related genes (**F**); Analysis relationships of immune cells in all samples and in MIRI samples only, the correlation analysis of immune cells of all samples is shown in red and blue, with red representing positive correlation and blue representing negative correlation. The correlation analysis of immune cells of only MIRI samples is represented by yellow and green, yellow represents positive correlation, and green represents negative correlation (**G**). Correlation between Sirt2 and Sirt 6 with immune cells, correlation analysis of immune cells of all samples (H), and MIRI samples (I) employing the Pearson coefficient method

By comparing gene expression profiles between MIRI samples and healthy controls samples, we aimed to identify differentially expressed genes (DEGs) associated with MIRI pathology. In the dataset GSE130217, we applied stringent criteria for DEG selection, namely an adjusted P value < 0.05 and an absolute log fold change (|logFC|) greater than or equal to 1.0. This analysis resulted in the identification of 1807 DEGs that exhibited significant differences between MIRI and normal samples (Fig. 1A, B). To further investigate the expression patterns of Sirt family genes (SFGs), we generated Fig. 1C to visualize their expression levels. Through the screening of differential expression, we ultimately identified five Sirt family regulators (Sirt2, Sirt3, Sirt4, Sirt5 and Sirt6) as differentially expressed Sirt family genes (DeSFGs). In MIRI tissues compared to normal samples, the expression of Sirt2, Sirt3, Sirt4, Sirt5 and Sirt6 was downregulated. Totally, 966 differentially expressed genes were used to perform GO and KEGG enrichment analyses. GO enrichment analysis revealed that differential expression genes (DEGs) were mainly enriched in cytokine-mediated signaling pathway (BP), receptor complex (CC), and cytokine (MF) et al. (Fig. 1D and Additional file 4). In the KEGG pathway, DEGs were enriched cytokine-cytokine receptor interaction, chemokine signaling pathway and ECM-receptor interaction et al. (Fig. 1E and Additional file 4). Next, we identified the two DeSFGs, named, Sirt2 and Sirt6, among the differentially expressed Sirt family genes, because these two genes have been closely associated with mitophagy and pyroptosis [24–26], especially Sirt6. To understand the relationship between these two DeSFGs and mitophagy or pyroptosis, we constructed an interaction network, as depicted in Fig. 1F. In this network, we observed an interaction between Sirt6 and SQSTM1 (p62), BECLIN1, and NLRP3 et al. After filtering out the immune cell types that were not present (0 value in more than 80% of samples), the remaining 13 types of immune cells were subject to Pearson correlation analysis, including the relationships of immune cells in all samples and in MIRI samples only (Fig. 1G). According to the expression of Sirt2 and Sirt6 and immune cell content in each sample, the correlation of was calculated by the Pearson correlation analysis (Fig. 1H, I).

### Exosome characterization and transfection efficiency

**Fig. 2:**
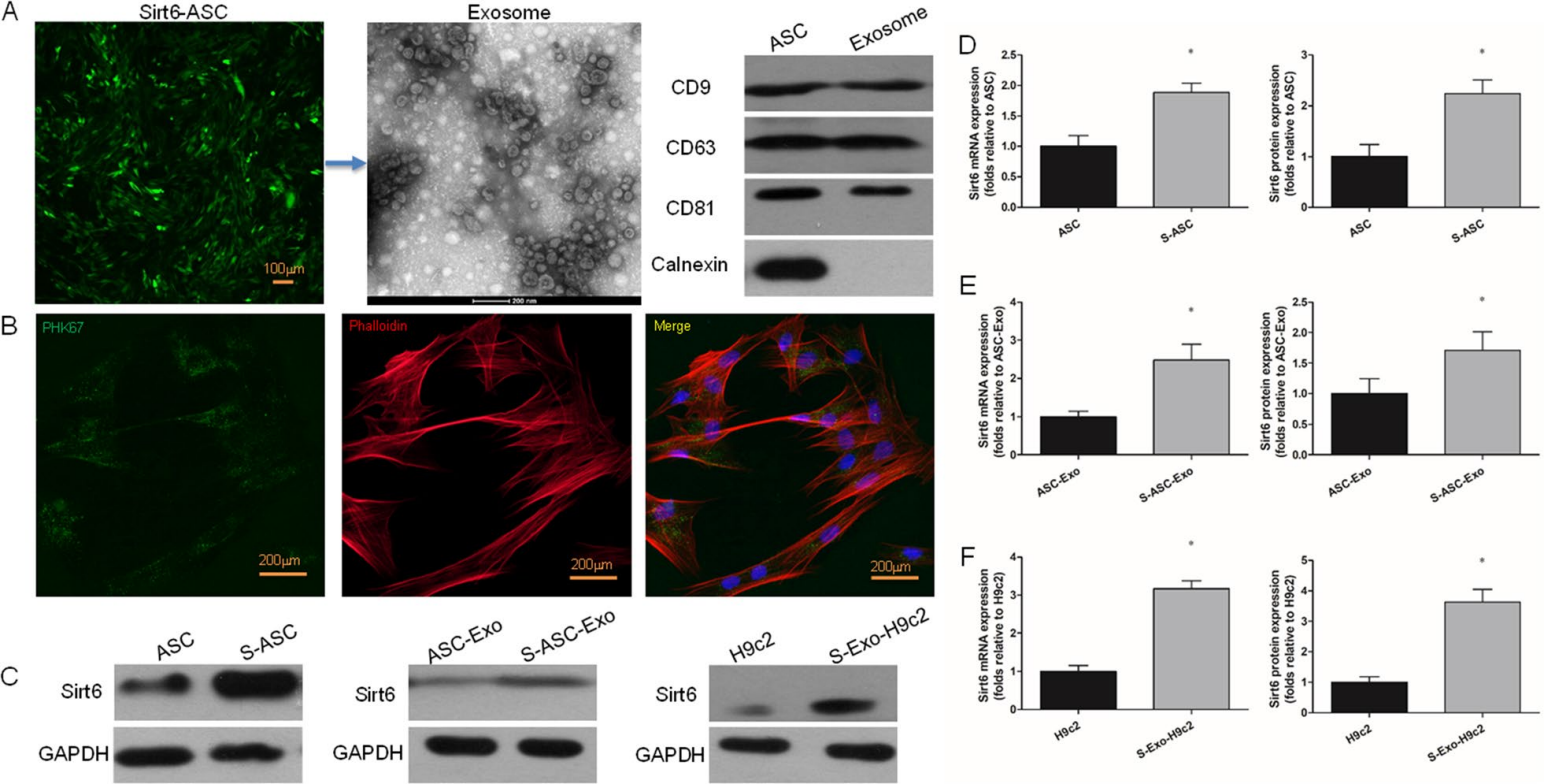
Exosome characterization and transfection efficiency. **A** ASCs transfected with pre-Sirt6 (green fluorescence). TEM image of exosome. Representative western blot pictures showing positive expressions of CD9, CD63, and CD81, while negative expression of Calnexin. **B** Representative image of PHK67-labeled exosomes (green) and Phalloidin (red) double-immunostaining in H9c2s. **C** Representative western blot pictures showing Sirt6 expressions. **D–F** Densitometric quantification of Sirt6 expression levels in transfected groups versus un-transfected groups. *P < 0.05. S-ASC, ASCs transfected with pre-Sirt6; ASC-Exo, exosomes from ASCs; S-ASC-Exo, exosomes from S-ASCs; S-Exo-H9c2, H9c2s engulfing S-ASC-Exo

As shown in Fig. 2A, ASCs transfected with pre-Sirt6 showed green fluorescence and exosomes from S-ASCs were discoid membrane vesicles. The surface markers CD9, CD63, and CD81 were positive, while Calnexin was negative. The PHK67-labeled exosomes were engulfed by H9c2s (Fig. 2B). Representative western blot pictures of Sirt6 in ASCs, exosomes, and H9c2s are shown in (Fig. 2C). After successfully obtaining Sirt6-enriched adipose stem cells (S-ASCs), we isolated S-ASC-derived exosomes (S-ASC-Exo) and performed additional characterization of the exosome cargo. The comprehensive characterization of exosomal cargo suggested that the Sirt6 transfection might have altered some of the cargo components, which could be responsible for the observed therapeutic effects of S-ASC-Exo in MIRI. Densitometric analysis (Fig. 2D–F) revealed that compared with un-transfected groups, transfection of Sirt6 markedly enhanced the expressions of Sirt6 mRNA and Sirt6 protein in S-ASCs, S-ASC-Exo, and H9c2s engulfing S-ASC-Exo.

### S-ASC-Exo inhibits AIM2-pyroptosis and promotes mitophagy in H9c2s subjected to AR

**Fig. 3:**
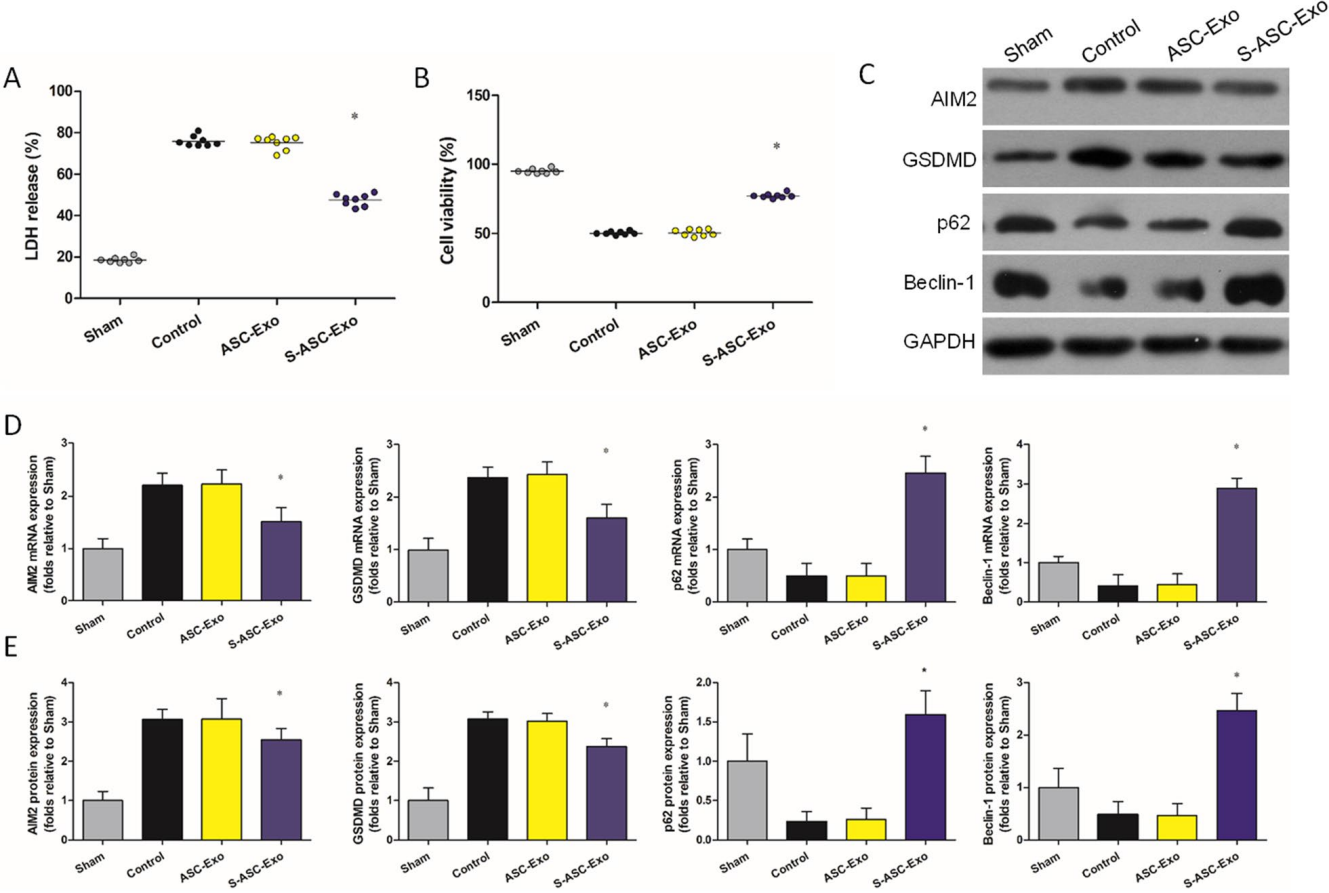
S-ASC-Exo inhibits AIM2-pyroptosis and promotes mitophagy in H9c2s subjected to AR. **A** LDH measured by ELSIA (*P* = 0.015). **B** Cell viability measured by MTT (*P* = 0.021). **C** Representative western blot pictures showing expressions of AIM2, GSDMD, p62, and Beclin-1. **D** mRNA levels of AIM2 (*P* = 0.018), GSDMD (*P* = 0.016), p62 (*P* = 0.004), and Beclin-1 (*P* = 0.007). **E** Densitometric quantification of AIM2 (*P* = 0.042), GSDMD (*P* = 0.045), p62 (*P* < 0.001), and Beclin-1 (*P* = 0.003) expressions. *P < 0.05, versus control

AR significantly increased the level of LDH in the control group (Fig. 3A). Level of LDH was lower in the S-ASC-Exo group. AR significantly decreased the cell viability in the control group (Fig. 3B). The cell viability was higher in the S-ASC-Exo group. Compared with the control, ASC-Exo did not affect the level of LDH and the cell viability. Results of qRT-PCR are summarized in Fig. 3D. The expressions of AIM2, GSDMD, p62, and Beclin-1 could be detected in the sham group. In H9c2s subjected to AR, the levels of AIM2 and GSDMD increased, but the levels of p62 and Beclin-1 decreased. Sirt6-enriched exosomes robustly decreased the levels of AIM2 and GSDMD, but increased the levels of p62 and Beclin-1. Compared with control, ASC-Exo did not affect the levels of AIM2, GSDMD, p62, and Beclin-1. Representative western blot pictures are shown in Fig. 3C. Densitometric analysis (Fig. 3E) revealed that Sirt6-enriched exosomes dramatically inhibited the expressions of AIM2 and GSDMD, but enhanced the expressions of p62 and Beclin-1. There was no significant difference in the expressions of AIM2, GSDMD, p62, and Beclin-1 between the control groups and the ASC-Exo groups.

### siSirt6 reverses the cardioprotective effect of S-ASC-Exo

**Fig. 4:**
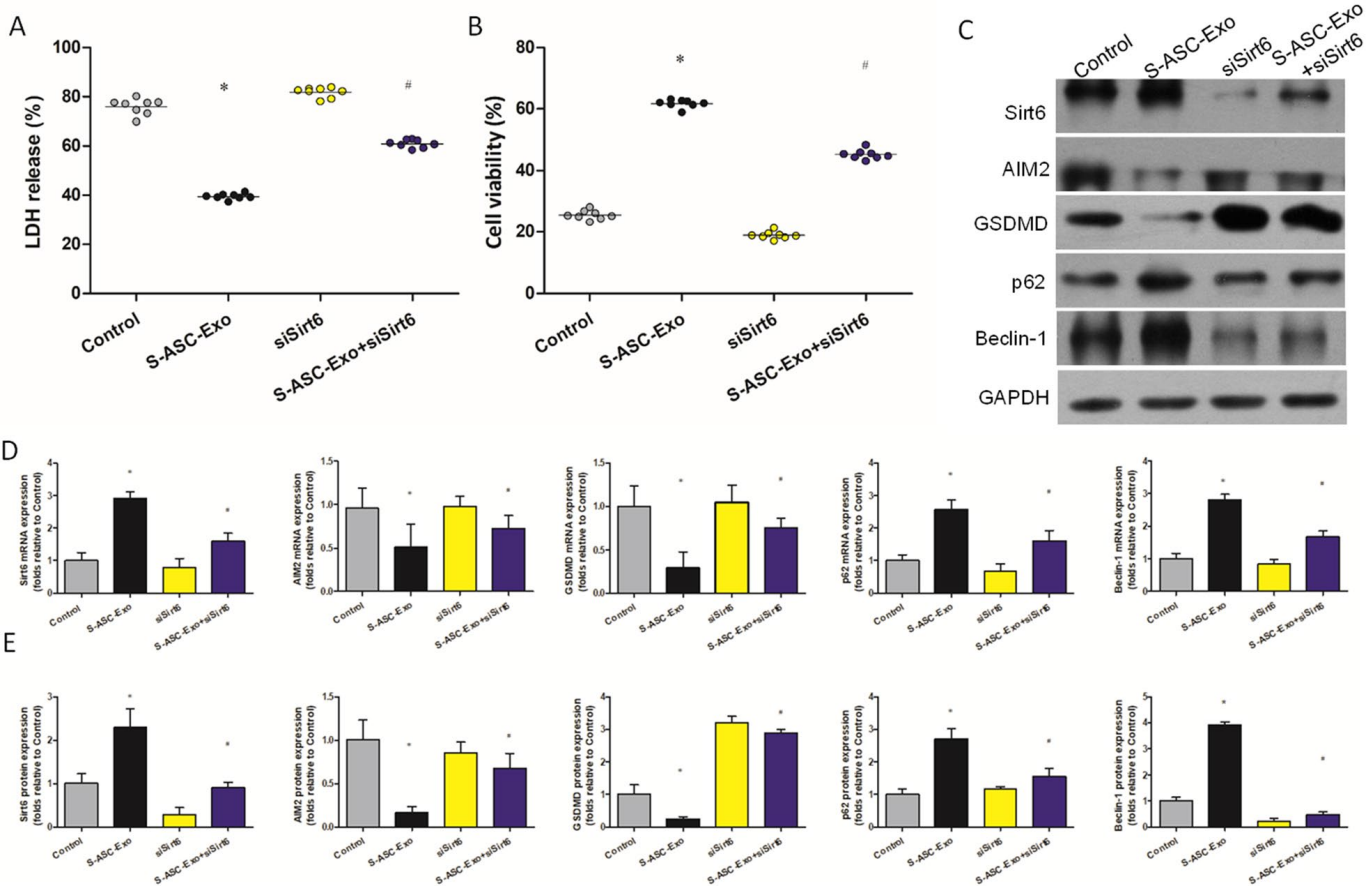
siSirt6 reverses the cardioprotective effect of S-ASC-Exo. **A** LDH measured by ELSIA (*P = 0.012, ^#^*P* = 0.025). **B** Cell viability measured by MTT (*P = 0.014, ^#^*P* = 0.033). **C** Representative western blot pictures showing expressions of Sirt6, AIM2, GSDMD, p62, and Beclin-1. **D** mRNA expressions of Sirt6 (*P = 0.024, ^#^*P* = 0.031), AIM2 (*P = 0.04, ^#^*P* = 0.045), GSDMD (*P = 0.015, ^#^*P* = 0.035), p62 (*P = 0.029, ^#^*P* = 0.036), and Beclin-1 (*P = 0.017, ^#^*P* = 0.027). **E** Densitometric quantification of Sirt6 (*P = 0.031, ^#^*P* = 0.038), AIM2 (*P = 0.001, ^#^*P* = 0.013), GSDMD (* *P* = 0.007, ^#^*P* < 0.001), p62 (*P = 0.021, ^#^*P* = 0.029), and Beclin-1 (*P = 0.003, ^#^*P* = 0.005) expressions. *P < 0.05, versus control, ^#^*P* < 0.05, versus S-ASC-Exo

Administration of siSirt6 did not affect the level of LDH and the cell viability. No significant difference was detected between the control group and siSirt6 group. Compared with S-ASC-Exo, S-ASC-Exo + siSirt6 increased the level of LDH (Fig. 4A) and decreased the cell viability (Fig. 4B). We used siSirt6 to investigate the molecular mechanism of S-ASC-Exo protecting against AR. Results of qRT-PCR are summarized in Fig. 4D. Compared with control, S-ASC-Exo significantly increased the levels of Sirt6, p62, and Beclin-1, but decreased the levels of AIM2 and GSDMD. Compared with S-ASC-Exo, S-ASC-Exo + siSirt6 decreased the levels of Sirt6, p62, and Beclin-1, but increased the levels of AIM2 and GSDMD. Representative western blot pictures are shown in Fig. 4C, and densitometric quantification of these protein expressions are summarized in Fig. 4E. Compared with control, S-ASC-Exo significantly enhanced the expressions of Sirt6, p62, and Beclin-1, but inhibited the expressions of AIM2 and GSDMD. Compared with S-ASC-Exo, S-ASC-Exo + siSirt6 inhibited the expressions of Sirt6, p62, and Beclin-1, but enhanced the expressions of AIM2 and GSDMD.

### Effect of VX-765 on the induction of pyroptosis in H9c2s subjected to AR

**Fig. 5:**
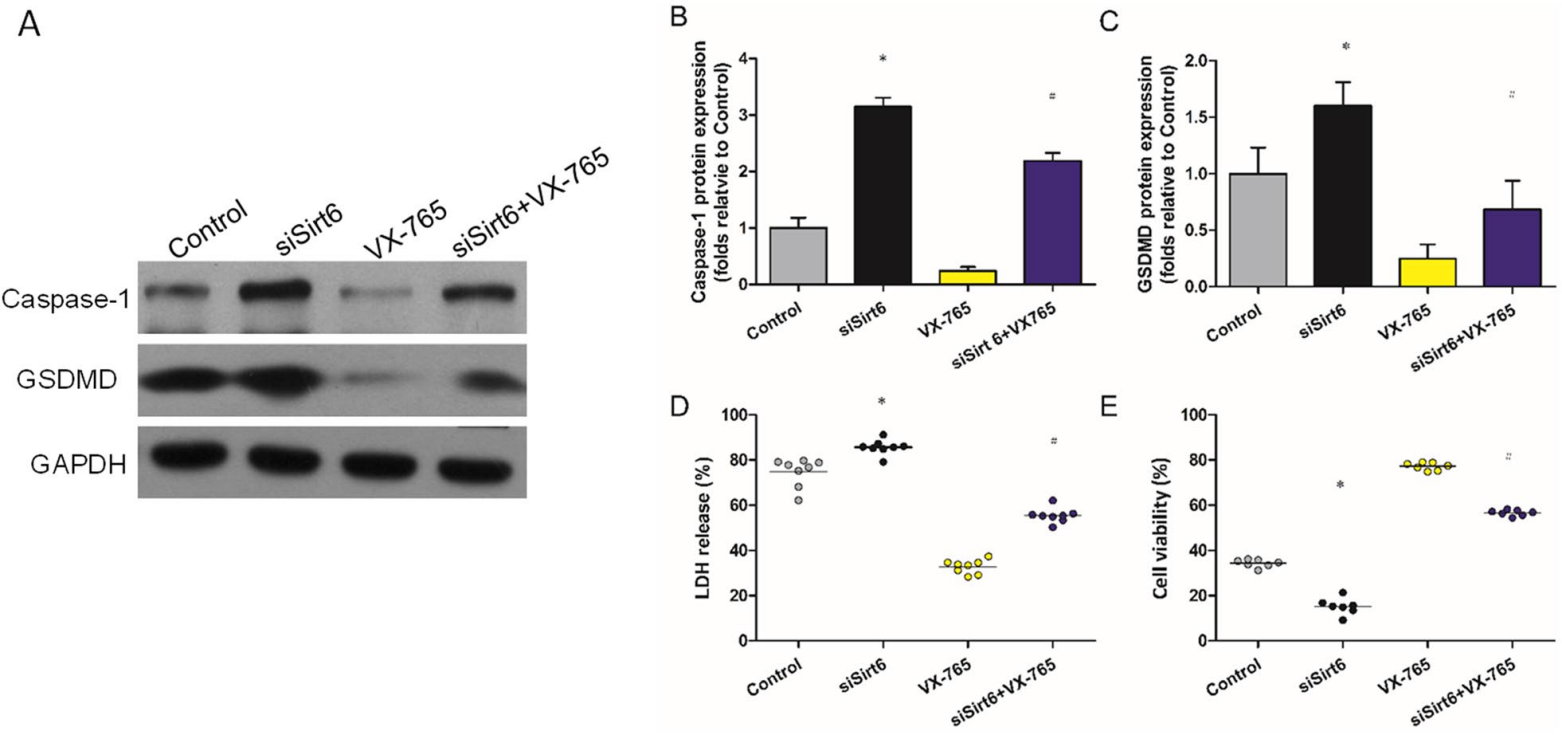
Effect of VX-765 on the induction of pyroptosis. **A** Representative western blot pictures showing Caspase-1 and GSDMD expressions. **B**, **C** Densitometric quantification of Caspase-1 (*P = 0.012, ^#^*P* = 0.037) and GSDMD (*P = 0.024, ^#^*P* = 0.013) expressions. **D** LDH measured by ELSIA (*P = 0.047, ^#^*P* = 0.026). (**E**) Cell viability measured by MTT (*P = 0.018, ^#^*P* = 0.006). *P < 0.05, versus control, ^#^*P* < 0.05, versus siSirt6

From the above results, siSirt6 plays a role in inducing pyroptosis. To further investigate the regulatory mechanism of Sirt6/pyroptosis in H9c2s subjected to AR, VX-765 (inhibitor of caspase-1) [27] was administered. Representative western blot pictures of Caspase-1 and GSDMD expressions are shown in Fig. 5A. The induction of pyroptosis by siSirt6 was partially inhibited by VX-765. Compared with siSirt6, the expressions of Caspase-1 and GSDMD in the siSirt6 + VX-765 group were decreased dramatically (Fig. 5B, C). Compared with control, siSirt6 increased the level of LDH and decreased the cell viability, which was reversed by VX-765 (Fig. 5D, E).

### Effect of 3-MA on the promotion of mitophagy in H9c2s subjected to AR

**Fig. 6:**
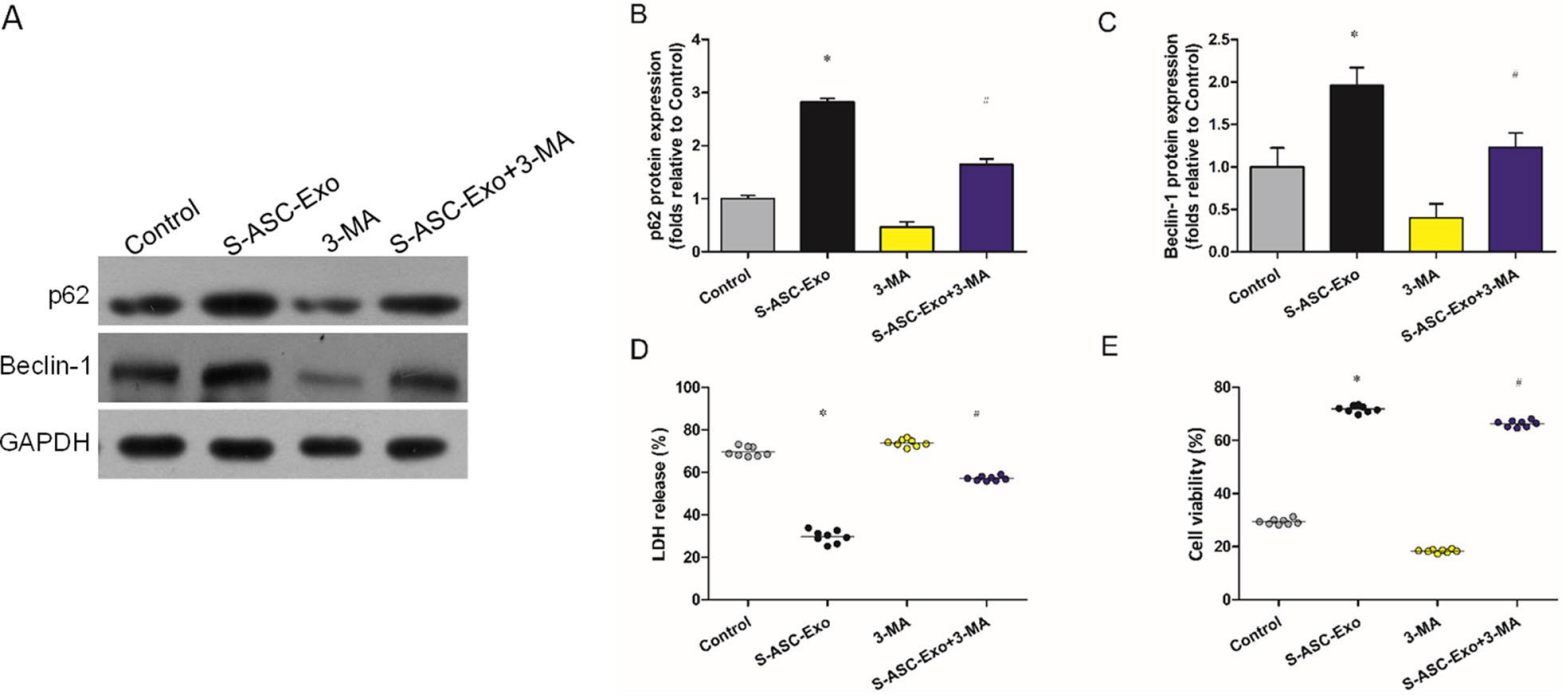
Effect of 3-MA on the induction of mitophagy. **A** Representative western blot pictures showing p62 and Beclin-1 expressions. **B**, **C** Densitometric quantification of p62 (*P = 0.027, ^#^*P* = 0.033) and Beclin-1 (*P = 0.035, ^#^*P* = 0.042) expressions. **D** LDH measured by ELSIA (*P = 0.008, ^#^*P* = 0.014). (**E**) Cell viability measured by MTT (*P = 0.005, ^#^*P* = 0.046). *P < 0.05, versus control, ^#^*P* < 0.05, versus S-ASC-Exo

From the above results, S-ASC-Exo plays a role in promoting mitophagy. To further investigate the regulatory mechanism of Sirt6/mitophagy in H9c2s subjected to AR, 3-MA (inhibitor of mitophagy) was administered. Representative western blot pictures of Caspase-1 and GSDMD expressions are shown in Fig. 6A. The promotion of mitophagy by S-ASC-Exo was partially inhibited by 3-MA. Compared with S-ASC-Exo, the expressions of p62 and Beclin-1 in the S-ASC-Exo + 3-MA group were decreased dramatically (Fig. 6B, C). Compared with control, S-ASC-Exo decreased the level of LDH and increased the cell viability, which was reversed by 3-MA (Fig. 6D, E).

### Ultrastructural and histologic examinations, and protein expressions in ischemic myocardia

**Fig. 7:**
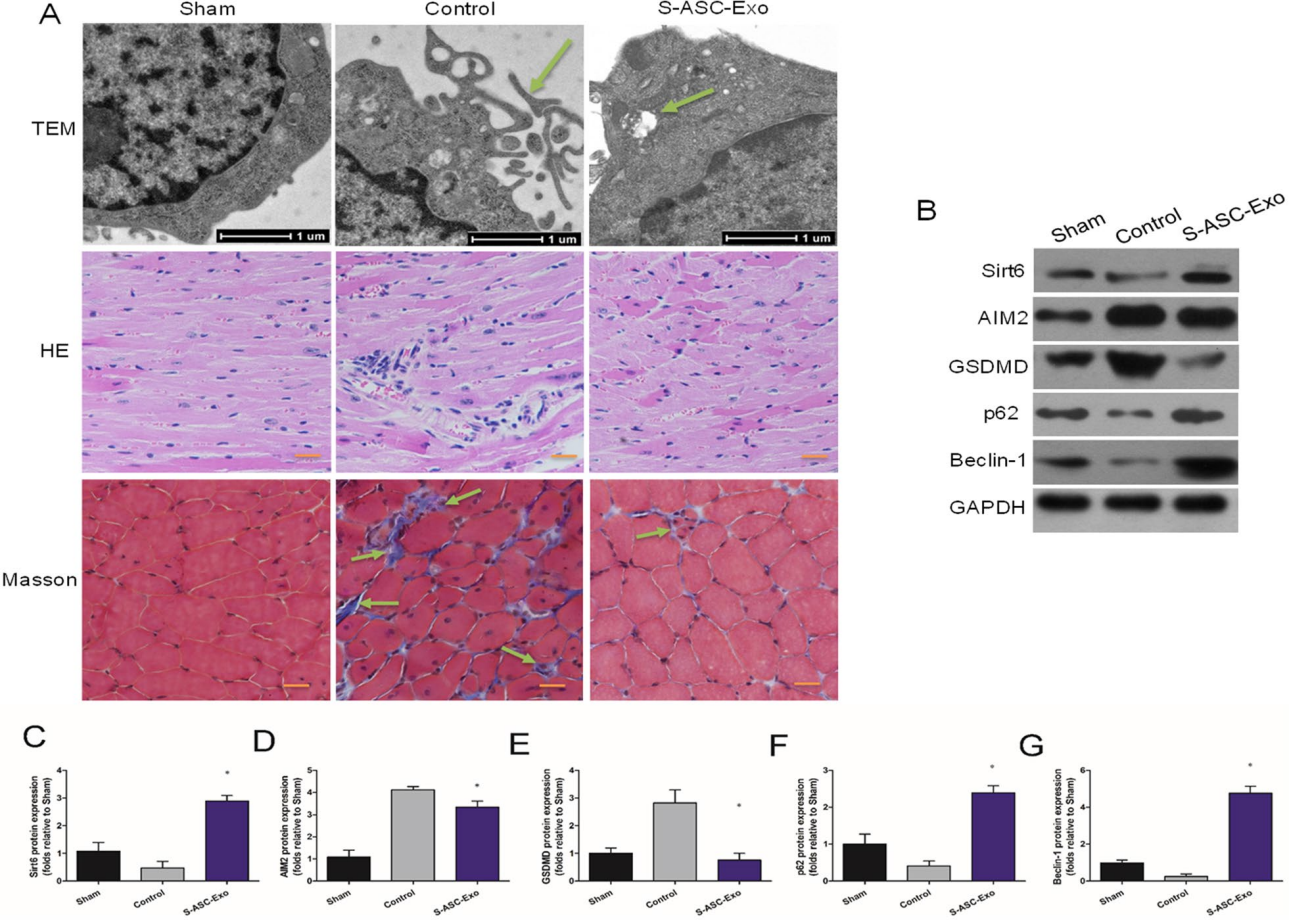
Ultrastructural and histologic examinations, and protein expressions in ischemic myocardia. **A** Representative TEM pictures, HE staining, and Masson staining sections of myocardial tissue. Green arrows indicating cell membrane lysis in the control group and typical mitophagy in the S-ASC-Exo group. For Masson staining, green arrows indicating fibrosis. **B** Representative western blot pictures showing expressions of Sirt6, AIM2, GSDMD, p62, and Beclin-1 in myocardial tissues. **C–G** Densitometric quantification of Sirt6 (*P* = 0.003), AIM2 (*P* = 0.041), GSDMD (*P* = 0.005), p62 (*P* = 0.004), and Beclin-1 (*P* < 0.001) expressions: sham (*n* = 6), control (*n* = 6), and S-ASC-Exo (*n* = 6) groups. *P < 0.05, versus control. Scale bar = 100 mm

Pyroptosis is characterized by the constant expansion of cell until the cell membrane breaks, allowing inflammatory cytokines to escape from cell membrane and then activating intense inflammatory reactions [7, 28]. As illustrated in TEM images (Fig. 7A), intact cell membrane was detected in the sham group. Amounts of cell membrane lysis were found in the control group. After administration of S-ASC-Exo, the cell membrane lysis was alleviated, and a typical mitophagy was verified by the swollen mitochondria encapsulated by autophagosome. Representative HE pictures (Fig. 7A) showed that severe myocardial damage was characterized by disordered arrangement, karyolysis, hyperchromatic cytoplasm, and inflammatory cell infiltration in the control group. Slighter histologic changes were detected in the ischemic myocardia treated with S-ASC-Exo. Masson trichome staining (Fig. 7A) revealed that compared with control, a reduced volume of collagen deposition and fibrotic lesion was detected in the S-ASC-Exo group. The expressions of AIM2-pyroptosis and mitophagy in ischemic myocardia were consistent with the western blot results at cellular level. Representative western blot pictures are shown in Fig. 7B. The expressions of Sirt6, AIM2-pyroptosis, and mitophagy could be detected in myocardia of sham group. Densitometric analysis revealed that compared with control, Sirt6-enriched exosomes markedly enhanced the expressions of Sirt6 (Fig. 7C), p62 (Fig. 7F), and Beclin-1 (Fig. 7G), while inhibited the expressions of AIM2 (Fig. 7D) and GSDMD (Fig. 7E).

### S-ASC-Exo improves cardiac function and reduces infract size

**Fig. 8:**
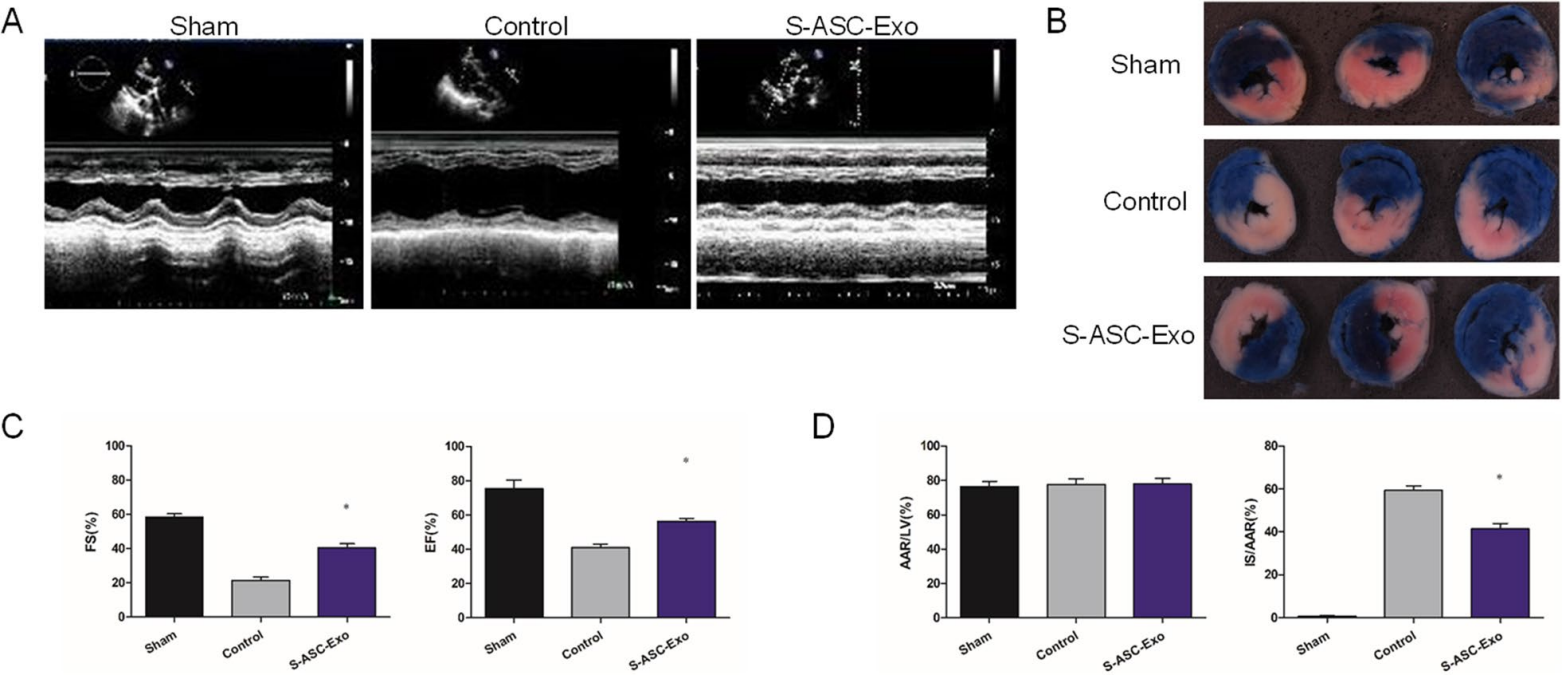
S-ASC-Exo improves cardiac function and reduces infract size. **A** M-mode echocardiography images. **B** Representative digital images of infracted tissue (pale) within the area at risk (red) and non-ischemic zones (blue). **C** Calculation of FS% (*P* = 0.013) and EF% (*P* = 0.022) among the sham (*n* = 6), control (*n* = 6), and S-ASC-Exo (*n* = 6) groups. **D** Calculations of IS (*P* = 0.029) and AAR of the sham (*n* = 6), control (*n* = 6), and S-ASC-Exo (*n* = 6) groups. *P < 0.05, versus control

Representative echocardiography images are shown in Fig. 8A. Results revealed that EF and FS were both decreased in the control group compared with that of the sham group, and administration of S-ASC-Exo significantly attenuated the impact (Fig. 8C). Representative images of cross sections of ischemic myocardia are shown in Fig. 8B. AAR/LV values did not differ significantly among the sham, control, and S-ASC-Exo groups. The IS/AAR of the control group was the highest, while administration of S-ASC-Exo reduced the IS/AAR (Fig. 8D).

## Discussion

The major findings of this study are summarized in Graphical abstract. The satisfactory cardioprotection of S-ASC-Exo against MIRI are associated with (1) decreased LDH release, (2) increased viability of cardiomyocyte, (3) enhanced mitophagy and inhibited AIM2-pyroptosis in vitro and in vivo, (4) improved cardiac function and reduced infarct size. In our study, we engineered Sirt6-expressing exosomes derived from adipose-derived stem cells (ASCs). These modified exosomes were enriched with Sirt6, a protein known to play a crucial role in cardiac protection. Through the incorporation of Sirt6 into exosomes, we believe that these engineered exosomes gained additional therapeutic properties, making them capable of improving myocardial ischemia–reperfusion injury.

Based on the ability to cross the vessel barrier, excellent biocompatibility, and low immunogenicity, exosomes have emerged as efficient nanoscale messengers for cells or cell-tissue communications [16, 29]. Mounting evidence suggests that exosomes possess great capacity to deliver protective non-coding RNAs [30, 31], miRNAs [32, 33], and healthy organelles [34] for the treatment of ischemic diseases. ASCs are abundant in source, easy to be harvested and modified, and can robustly release exosomes, which would meet huge clinical demands in future [18, 35]. Sirt6 mainly functions through binding its NAD + domains to corresponding protein to promote deacetylation, which leads to beneficial regulations in pathophysiological processes including DNA repair, inflammation control, inhibition of oxidative stress, and autophagy [36]. We revealed that ASCs can be genetically modified to overexpress Sirt6, and exosomes from Sirt6-ASCs in ischemic myocardia were associated with anti-pyroptotic effects, induction of mitophagy and improved cardiac outcomes. However, paradoxical results have been drawn. Overexpression of Sirt6 has been shown to induce increased ROS production and decreased cell viability under oxidative stress in neurons [37]. In our study, we observed that S-ASC-Exo exhibited a markedly stronger cardioprotective effect than ASC-Exo, as evidenced by various functional parameters in both in vitro and in vivo models of MIRI. In our study, we found that S-ASC-Exo exhibited pronounced cardioprotective effects in MIRI through the enhancement of mitophagy and the inhibition of AIM2 inflammasome-mediated pyroptosis. In comparing our findings with relevant previous studies, our research aligns with the observations of a study demonstrated a significant reduction in infarct size following treatment with adipose stem cell-derived exosomes in a rat model of myocardial infarction [38], supporting the potential therapeutic role of exosomes in cardiac protection. Similarly, Sun et al. investigated the effects of exosomes derived from mesenchymal stem cells in reducing myocardial ischemia–reperfusion injury, reporting improved cardiac function [39]. Our findings further expand our understanding of the multifaceted pathways implicated in mitigating myocardial ischemia–reperfusion injury.

Although our focus was on the role of Sirt6, one cannot overlook the potential contribution of other exosome cargo components in mediating the observed therapeutic effect. It is plausible that alterations in the proteomic, lipidomic, and nucleic acid content resulting from the Sirt6 transfection enriched the therapeutic properties of S-ASC-Exo. Thus, future investigations on the specific cargo changes and their functional implications are warranted to gain more comprehensive insights into the therapeutic effects of S-ASC-Exo in MIRI. This finding strongly suggests that the exogenous overexpression of Sirt6 in adipose stem cells plays a significant role in the observed beneficial effects. Furthermore, siRNA-mediated Sirt6 silencing reversed the cardioprotective effects of S-ASC-Exo, strongly supporting the direct involvement and crucial role of Sirt6 in driving these benefits. In other conditions, Sirt6 functions according different disease contexts. Several studies have confirmed Sirt6 as both an oncogenic factor [40] and a tumor suppressor in some cancers [41]. Each member of Sirtuin family has a series of target proteins, and the functions of target proteins may be decided by the pathological conditions, intensity of stimuli, time point of intervention, and the effector organ, which may result in the inconsistency of the final outcome of regulation of Sirt6.

To elucidate the potential mechanisms of cardioprotective effects induced by overexpression of Sirt6 against AR, possibilities for the target proteins were explored. The molecular interaction between Sirt6 and AIM2-pyroptosis forms a complex signal transduction pathway to control the occurrence and outcome of inflammation. Sirt6 is indicated to be an endogenous inhibitive regulator of oxidative stress [42], and oxidative stress directly activate the AIM2 and its downstream signaling pathway [43]. AIM2 pattern recognition receptor binds to infectious and/or non-infectious stimuli, which activates the downstream multi-protein inflammasome complex, and then promotes caspase-1 dependent GSDMD to locate on cell membrane, forming pores conducive to the release of IL-1β and IL-18, and finally leads to pyroptosis [43]. Through negatively regulating the Lin28/Let7 signaling pathway, overexpression of Sirt6 decreased GSDMD cleavage and pyroptosis in endothelial cells, which retarded the progression of atherosclerosis [44]. Another study indicated that Sirt6-induced autophagy restricted TREM-1-mediated pyroptosis, which renders Sirt6 and TREM-1 inflammatory markers for use in prognosis stratification of acute myocardial infarction [45]. However, it is uncertain what role Sirt6 plays during pyroptosis of cardiomyocytes subjected to AR. We revealed that in H9c2s subjected to AR, decreased level of Sirt6 and elevated levels of AIM2 and GSDMD were reversed by exosomes from ASCs overexpressing Sirt6, suggesting that S-ASC-Exo may account for cardioprotective effects via anti-AIM2-pyroptosis. It is worth noting that compared with control, the LDH release decreased and the cell viability increased. On the contrary, the AIM2-pyroptosis was enhanced after administration of siRNA-Sirt6, and improved cell survival was abolished by siRNA-Sirt6. Moreover, the enhanced AIM2-pytoptosis induced by siRNA-Sirt6 was abolished partially by the antagonist of capase-1 inhibitor VX765, also known as pyroptosis inhibitor. Hence, it is likely that the Sirt6-AIM2-GSDMD signaling pathway is involved in the anti-pyroptotic and cardioprotective effects conducted by upregulation of Sirt6.

Mitophagy is a selective autophagic process specifically designed for the removal and recycle of damaged or unneeded mitochondria from a cell, which reduces cell death and sustains cellular activity [4]. Evidences indicate that mitophagy broadly participates in regulation of pathophysiological processes of cardio-cerebral diseases, especially oxidative stress. For example, dysfunction in mitophagy is associated with neurodegenerative diseases such as Huntington [46], Parkinson [47], and Alzheimer [48]. Abnormality of mitophagy induces LDL accumulation and mitochondrial dysfunction, thereby aggravating diabetic cardiomyopathy [49]. In the context of ischemia, mitophagy plays important roles in cardiac ischemia and protects against cardiac injury by mitochondrial clearance [50]. Mitophagy also is involved in the cardioprotection against acute ischemic injury by inhibition of Nrf2 and endoplasmic reticulum stress [51]. Mitochondria are the powerhouse and overproduce ROS after ischemia, which induces cell death through the release of proapoptotic and pro-pyroptotic proteins such as Caspase-1 [52]. Although the mechanisms of cell death after cardiac ischemia remain unclear, mitochondria obviously activate mitochondria-dependent pyroptosis signaling pathways [53]. Consistently, the expressions of mitophagy and the cardiomyocyte survival were enhanced by administration of S-ASC-Exo in the current study, and the improvements were reversed by siRNA-Sirt6 and mitophagy inhibitor 3-MA, suggesting that the mitophagy was involved in mediating the cardioprotection of overexpression of Sirt6. Excellent ultrastructural and histologic results showed that mitophagy inhibited the pyroptotsis-induced membrane lysis, myocardial fibrosis, and severe histological changes. Another important finding was that Sirt6 transferred to ischemic myocardia by Sirt6 enriched exosomes inhibited the expressions of AIM2-pyroptosis, enhanced the expressions of mitophagy, improved cardiac functions, and reduced infarct size. Taken together, it is plausible that Sirt6 is the mediator of both AIM2-pyroptosis and mitophagy, and conducts cardiopretection against ischemia/reperfusion injury, which can be enhanced by genetically modified adipose stem cells to overload Sirt6.

Several limitations of this study merit concern. On the one hand, excessive mitophagy in myocardial ischemia may promote loss of normal myocytes [54] and suppressing excessive mitophagy also has been shown to mediate cardioprotection [55]. Excessive depletion of mitochondria by mitophagy may lead to energy shortage and cellular side injuries. On the other hand, it is not known whether other cardioprotective effects rather than anti-pyroptotic and pro-mitophagy effects contribute to the cardioprotection. The mechanism by which Sirt6 regulates AIM2-pyroptosis and mitophagy under MIRI have not fully investigated. Mitochondrial probes might be used to investigate the autophagic machinery. Simultaneously, regarding the mechanism of S-ASC-Exo-induced mitophagy, it is important to note that our study primarily focused on the expression levels of AIM2, GSDMD, p62, and Beclin-1 as potential markers of mitophagy initiation. However, we acknowledge that this sole expression analysis may not provide a comprehensive understanding of the underlying molecular pathways involved in mitophagy induction. We were unable to investigate other potential mechanisms of mitophagy induction, such as the PINK1/Parkin pathway or receptor-mediated mitophagy involving BNIP3, NIX, PHB2, and FUNDC1 in the context of S-ASC-Exo treatment. Future studies should consider exploring these pathways to gain a more comprehensive understanding of S-ASC-Exo-induced mitophagy. Last but not least, we do not identify the localization of S-ASC-Exo in vivo tissue, meanwhile, other regulators in ASC-exosome, aside from Sirt6, also conduct cardioprotective effects against ischemia/reperfusion injury, which needs to be further investigated. Despite these limitations, our study contributes to the understanding of the therapeutic potential of S-ASC-Exo in myocardial ischemia–reperfusion injury and sheds light on the involvement of pyroptosis and mitochondrial quality control. These findings provide a basis for future studies to explore the intricate molecular mechanisms of S-ASC-Exo-mediated effects, including mitophagy induction.

In a clinical context, these findings open up promising new avenues for the treatment of ischemia–reperfusion injury and related cardiovascular conditions. Exosome therapy, specifically leveraging exosomes derived from Sirt6-enriched adipose stem cells, appears to have a potent cardioprotective effect. As such, direct administration of these Sirt6-enriched exosomes can potentially serve as a noninvasive therapeutic option for patients suffering from heart disease. Through inhibiting AIM2-pyroptosis and promoting mitophagy in cardiomyocytes, the treatment could ensure the overall maintenance and recovery of the heart function after ischemia–reperfusion events. However, a potential challenge in this approach lies in the standardization and control of exosome enrichment and treatment delivery. More detailed studies are also required to investigate the long-term effects and potential side effects of this treatment. Furthermore, while our research was conducted on rat models, human trials are still necessary to cross-verify the efficacy and safety of this technique before it can be commercially introduced into clinical practice. Overall, our study signifies a critical turning point in understanding the regulation of AIM2-pyroptosis and mitophagy under ischemia–reperfusion conditions, supporting the exploration of Sirt6-enriched adipose stem cell-derived exosomes as a practical clinical treatment.

## Conclusion

In conclusion, our study sheds light on the novel therapeutic potential of Sirt6-enriched exosomes derived from adipose stem cells (ASCs) against myocardial ischemia/reperfusion injury (MIRI). Our work reveals that these exosomes attenuate the inflammatory cell death process, known as AIM2-mediated pyroptosis, while augmenting mitophagy, a protective process that eliminates damaged mitochondria in H9c2 cells. The administration of these exosomes in MIRI models resulted in improvements in cardiac function and reductions in infarct size, indicating their significant therapeutic potential. We provide novel insights into pyroptosis and mitophagy regulation by these exosomes, notably through the use of the caspase-1 inhibitor, VX-765. However, we caution that an over-induction of mitophagy could pose potential risks, thereby necessitating balanced therapeutic strategies. Furthermore, our study signifies the vast, unexplored potential of other members of the Sirtuin family, encouraging further inquiry into their roles in MIRI and other cardiac conditions. Our findings lay a robust foundation for future investigations into this innovative and promising therapeutic approach against MIRI. We hope that this research will pave the way for greater subsequent strides in the understanding and treatment of cardiac ischemia/reperfusion injury.

## Supplementary Information

**Additional file 1**. Sirt family effects and deficiency.

**Additional file 2**. Mitophagy-related genes (MRGs).

**Additional file 3**. Pyroptosis-related genes (PRGs).

**Additional file 4**. The bubble plot for GO enrichment analysis (A); The barplot for GO enrichment analysis (B) and the barplot for KEGG pathway enrichment analysis (C) for differential expression genes (DEGs).

## Data Availability

The datasets generated during and/or analyzed during the current study are available from the corresponding author on reasonable request. All data generated or analyzed during this study are included in this published article. Materials described in the manuscript, including all relevant raw data, will be freely available to any scientist wishing to use them for non-commercial purposes, without breaching participant confidentiality.
