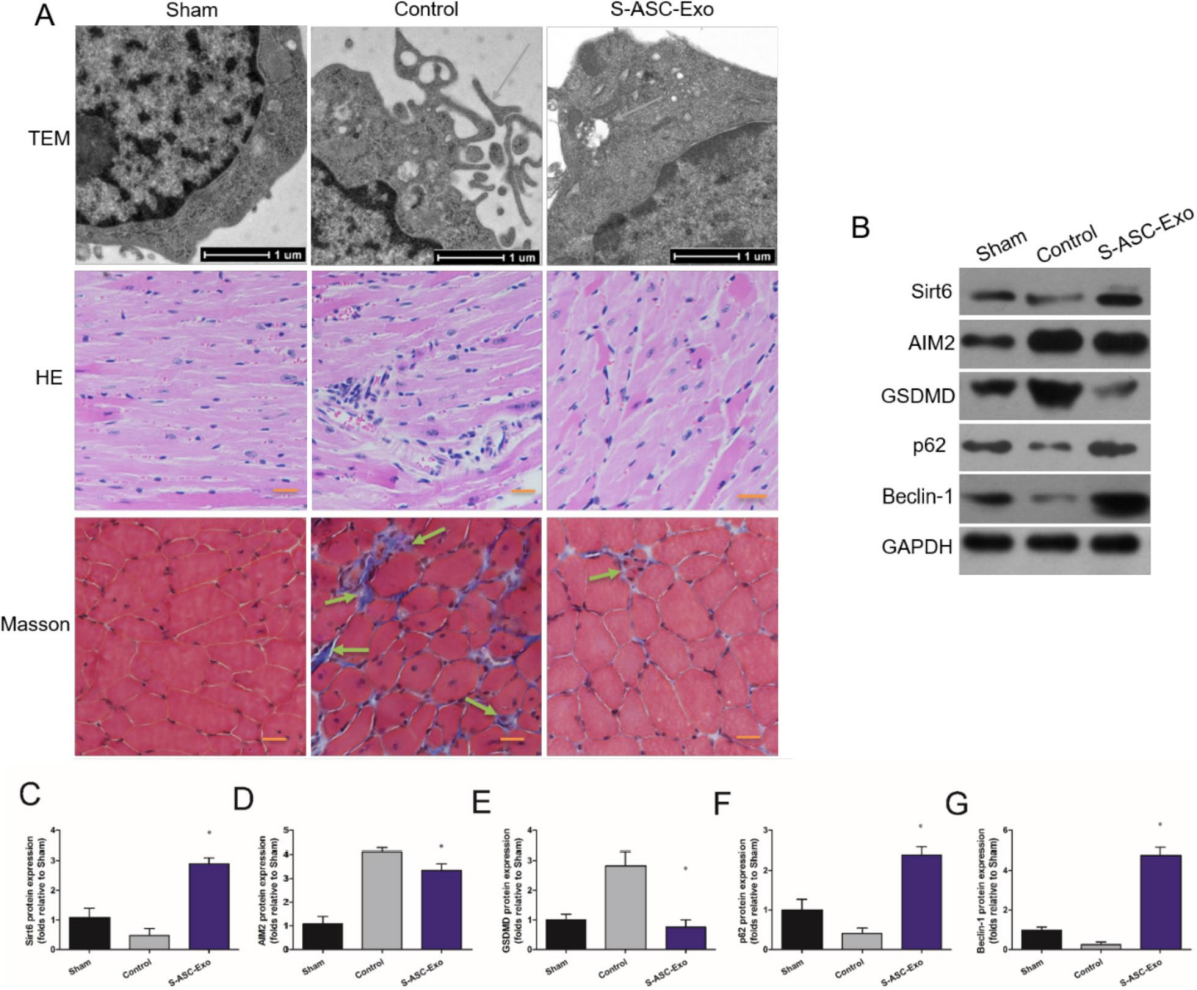# Correction: Exploring the therapeutic potential of Sirt6-enriched adipose stem cell-derived exosomes in myocardial ischemia–reperfusion injury: unfolding new epigenetic frontiers

**DOI:** 10.1186/s13148-024-01796-7

**Published:** 2024-12-20

**Authors:** Kun Liu, Hecheng Wang, Yiou Wang, Xiaoxu Zhang, Ruihu Wang, Zhaoxuan Zhang, Jian Wang, Xinran Lu, Xiaoyu Wu, Yanshuo Han

**Affiliations:** 1 Department of Cardiac Surgery, Affiliated Hospital, Guizhou Medical University, Guiyang, China; 2 School of Life and Pharmaceutical Sciences, Dalian University of Technology, Panjin, China; 3 Department of Anesthesiology, General Hospital of Northern Theater Command, Shenyang, China; 4 Department of Vascular Surgery, Shanghai Ninth People’s Hospital, Shanghai Jiao Tong University School of Medicine, Shanghai, China

**Correction: Clinical Epigenetics (2024) 16:7** 10.1186/s13148-023-01618-2

In the original publication of this article [1], an error was identified in Fig. 7A. Specifically, the Hematoxylin and Eosin (HE) staining image for the S-ASC-Exo group was incorrectly placed during figure assembly. This has been corrected by providing the accurate HE staining image for the S-ASC-Exo group. The incorrect and correct Fig. 7 are shown in this correction article.

**Incorrect Fig. 7**:
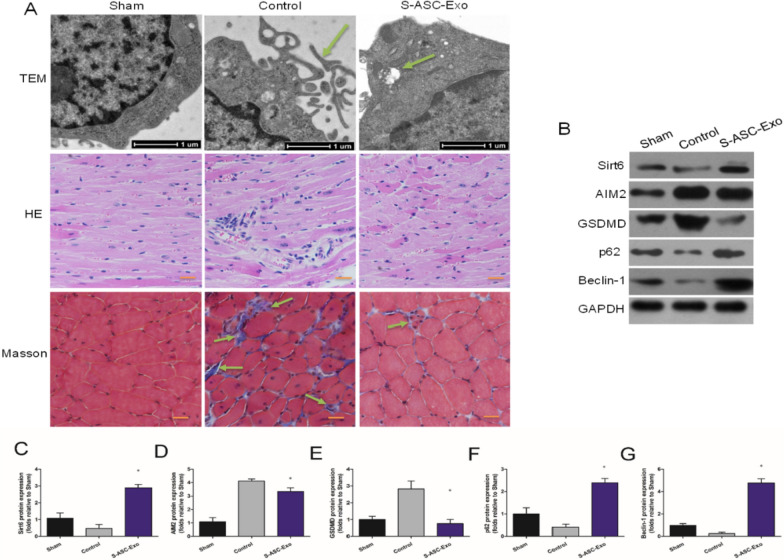


**Corrected Fig. 7**: