# Supplementary material for: Exploring the therapeutic potential of Sirt6-enriched adipose stem cell-derived exosomes in myocardial ischemia–reperfusion injury: unfolding new epigenetic frontiers
Source: Clin Epigenetics. 2024 Jan 3;16:7. doi: 10.1186/s13148-023-01618-2 (PMC10765803; doi:10.1186/s13148-023-01618-2)
Supplement: Supplementary file 1 — Additional file 1. Sirt family effects and deficiency. [file 13148_2023_1618_MOESM1_ESM.docx]

Table S1 Sirt family effects and deficiency

| Sirtuin | Histone modifications | Effects | Deficiency |
| --- | --- | --- | --- |
| SIRT1 | H4-K16dac | Transcriptional silencing, genome stability | Genomic instability, DNA damage accumulation in case of co-existing genotoxic stress |
|  | H3-K9dac |  |  |
|  | H1-K26dac |  |  |
|  | H3-K14dac | Transcriptional silencing |  |
|  | H-K16dac | Transcriptional silencing |  |
|  | H3-K56dac | Cell-cycle regulation, DNA repair |  |
|  | H3-K79me3 ^a^ | Genome stability ^a^ | Accelerated cellular aging ^a^ |
|  | H3-K27me3 ^a^ |  |  |
| SIRT2 | H3-K56dac | Chromatin condensation mitosis, DNA repair | Cell cycle inhibition at G2/M checkpoint, loss of histones at specific subtelomeric regions |
|  | H4-K16dac |  |  |
|  | H4K20me1 ^a^ | Genome stability,  S-phase progression ^a^ | Genomic instability and chromosomal aberrations, tumorigenesis ^a^ |
| SIRT3 | H4-K16dac ^a^ | Chromatin silencing, transcriptional silencing of stress-genes, DNA repair ^a^ | DNA damage ^a^ |
| SIRT6 | H3-K9dac | Protection of subtelomeric chromatin, | Aging-associated degenerative diseases, |
|  |  | Promoting DNA repair and genome stability, | Inflammaging, excessive cell apoptosis, lethal hypoglycemia |
|  | H3-K56dac | Prevention of aging and neurodegenerative diseases via silencing the NF-κB promotor sites |  |
| SIRT7 | H3-K18dac | Facilitation of DSB repair through NHEJ, | Proliferation stop, accelerated organismal aging, |
|  |  | Promotion of genomic stability, Induction of rRNA transcription | DNA damage, impaired glucose and lipid metabolism |

^a^ Indirect modification.
