# Supplementary material for: Exploring the therapeutic potential of Sirt6-enriched adipose stem cell-derived exosomes in myocardial ischemia–reperfusion injury: unfolding new epigenetic frontiers
Source: Clin Epigenetics. 2024 Jan 3;16:7. doi: 10.1186/s13148-023-01618-2 (PMC10765803; doi:10.1186/s13148-023-01618-2)
Supplement: Supplementary file 2 — Additional file 2. Mitophagy-related genes (MRGs). [file 13148_2023_1618_MOESM2_ESM.docx]

**Table S2.** Mitophagy-related genes (MRGs)

| No. | id |
| --- | --- |
| 1 | ATG12 |
| 2 | ATG5 |
| 3 | BECLIN1 |
| 4 | CSNK2A1 |
| 5 | CSNK2A2 |
| 6 | CSNK2B |
| 7 | FUNDC1 |
| 8 | MAP1LC3A |
| 9 | MAP1LC3B |
| 10 | MFN1 |
| 11 | MFN2 |
| 12 | MTERF3 |
| 13 | PGAM5 |
| 14 | PINK1 |
| 15 | PRKN |
| 16 | RPS27A |
| 17 | SQSTM1 |
| 18 | SRC |
| 19 | TOMM20 |
| 20 | TOMM22 |
| 21 | TOMM40 |
| 22 | TOMM5 |
| 23 | TOMM6 |
| 24 | TOMM7 |
| 25 | TOMM70 |
| 26 | UBA52 |
| 27 | UBB |
| 28 | UBC |
| 29 | ULK1 |
| 30 | VDAC1 |
