# Supplementary material for: Exploring the therapeutic potential of Sirt6-enriched adipose stem cell-derived exosomes in myocardial ischemia–reperfusion injury: unfolding new epigenetic frontiers
Source: Clin Epigenetics. 2024 Jan 3;16:7. doi: 10.1186/s13148-023-01618-2 (PMC10765803; doi:10.1186/s13148-023-01618-2)
Supplement: Supplementary file 3 — Additional file 3. Pyroptosis-related genes (PRGs). [file 13148_2023_1618_MOESM3_ESM.docx]

**Table S3.** Pyroptosis-related genes (PRGs)

| No. | Genes | Full-names |
| --- | --- | --- |
| 1 | BAK1 | BCL2 Antagonist/Killer 1 |
| 2 | BAX | BCL2 Associated X, Apoptosis Regulator |
| 3 | CASP1 | Caspase 1 |
| 4 | CASP3 | Caspase 3 |
| 5 | CASP4 | Caspase 4 |
| 6 | CASP5 | Caspase 5 |
| 7 | CHMP2A | Charged Multivesicular Body Protein 2A |
| 8 | CHMP2B | Charged Multivesicular Body Protein 2B |
| 9 | CHMP3 | Charged Multivesicular Body Protein 3 |
| 10 | CHMP4A | Charged Multivesicular Body Protein 4A |
| 11 | CHMP4B | Charged Multivesicular Body Protein 4B |
| 12 | CHMP4C | Charged Multivesicular Body Protein 4C |
| 13 | CHMP6 | Charged Multivesicular Body Protein 6 |
| 14 | CHMP7 | Charged Multivesicular Body Protein 7 |
| 15 | CYCS | Cytochrome C, Somatic |
| 16 | ELANE | Elastase, Neutrophil Expressed |
| 17 | GSDMD | Gasdermin D |
| 18 | GZMB | Granzyme B |
| 19 | HMGB1 | High Mobility Group Box 1 |
| 20 | IL18 | Interleukin 18 |
| 21 | IL1A | Interleukin 1 Alpha |
| 22 | IL1B | Interleukin 1 Beta |
| 23 | IRF1 | Interferon Regulatory Factor 1 |
| 24 | IRF2 | Interferon Regulatory Factor 2 |
| 25 | TP53 | Tumor Protein P53 |
| 26 | TP63 | Tumor Protein P63 |
| 27 | NLRP3 | NLR Family Pyrin Domain Containing 3 |
| 28 | GSDMB | Gasdermin B |
| 29 | AIM2 | Absent In Melanoma 2 |
| 30 | CASP6 | Caspase 6 |
| 31 | CASP8 | Caspase 8 |
| 32 | CASP9 | Caspase 9 |
| 33 | GPX4 | Glutathione Peroxidase 4 |
| 34 | GSDMA | Gasdermin A |
| 35 | GSDMC | Gasdermin C |
| 36 | GSDME | Gasdermin E |
| 37 | IL6 | Interleukin 6 |
| 38 | NLRC4 | NLR Family CARD Domain Containing 4 |
| 39 | NLRP1 | NLR Family Pyrin Domain Containing 1 |
| 40 | NLRP2 | NLR Family Pyrin Domain Containing 2 |
| 41 | NLRP6 | NLR Family Pyrin Domain Containing 6 |
| 42 | NLRP7 | NLR Family Pyrin Domain Containing 7 |
| 43 | NOD1 | Nucleotide Binding Oligomerization Domain Containing 1 |
| 44 | NOD2 | Nucleotide Binding Oligomerization Domain Containing 2 |
| 45 | PLCG1 | Phospholipase C Gamma 1 |
| 46 | PRKACA | Protein Kinase CAMP-Activated Catalytic Subunit Alpha |
| 47 | PYCARD | PYD And CARD Domain Containing |
| 48 | SCAF11 | SR-Related CTD Associated Factor 11 |
| 49 | TIRAP | TIR Domain Containing Adaptor Protein |
| 50 | TNF | Tumor Necrosis Factor |
| 51 | PJVK | Pejvakin |
