# Supplementary material for: Exploring the therapeutic potential of Sirt6-enriched adipose stem cell-derived exosomes in myocardial ischemia–reperfusion injury: unfolding new epigenetic frontiers
Source: Clin Epigenetics. 2024 Jan 3;16:7. doi: 10.1186/s13148-023-01618-2 (PMC10765803; doi:10.1186/s13148-023-01618-2)
Supplement: Supplementary file 4 — Additional file 4. The bubble plot for GO enrichment analysis (A); The barplot for GO enrichment analysis (B) and the barplot for KEGG pathway enrichment analysis (C) for differential expression genes (DEGs). [file 13148_2023_1618_MOESM4_ESM.docx]

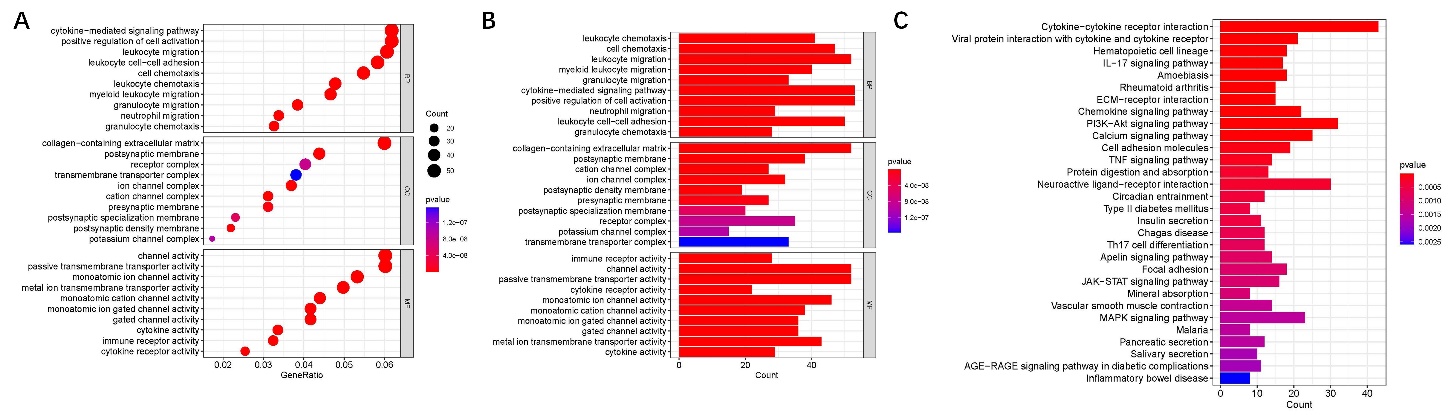


Figure S1. The bubble plot for GO enrichment analysis (A); The barplot for GO enrichment analysis (B) and the barplot for KEGG pathway enrichment analysis (C) for differential expression genes (DEGs).
